# Correlative microscopy of the constituents of a dinosaur rib fossil and hosting mudstone: Implications on diagenesis and fossil preservation

**DOI:** 10.1371/journal.pone.0186600

**Published:** 2017-10-19

**Authors:** Jung-Kyun Kim, Yong-Eun Kwon, Sang-Gil Lee, Chang-Yeon Kim, Jin-Gyu Kim, Min Huh, Eunji Lee, Youn-Joong Kim

**Affiliations:** 1 Graduate School of Analytical Science and Technology, Chungnam National University, Daejeon, South Korea; 2 Electron Microscopy Research Center, Korea Basic Science Institute, Daejeon, South Korea; 3 Korea Dinosaur Research Center, Chonnam National University, Gwangju, South Korea; 4 Department of Earth Systems and Environmental Sciences, Chonnam National University, Gwangju, South Korea; Seoul National University College of Medicine, REPUBLIC OF KOREA

## Abstract

We have applied correlative microscopy to identify the key constituents of a dorsal rib fossil from *Koreanosaurus boseongensis* and its hosting mudstone discovered at the rich fossil site in Boseong, South Korea, to investigate the factors that likely contributed to diagenesis and the preservation of fossil bone. Calcite and illite were the commonly occurring phases in the rib bone, hosting mudstone, and the boundary region in-between. The boundary region may have contributed to bone preservation once it fully formed by acting as a protective shell. Fluorapatite crystals in the rib bone matrix signified diagenetic alteration of the original bioapatite crystals. While calcite predominantly occupied vascular channels and cracks, platy illite crystals widely occupied miniscule pores throughout the bone matrix. Thorough transmission electron microscopy (TEM) study of illite within the bone matrix indicated the solid-state transformation of 1M to 2M without composition change, which was more evident from the lateral variation of 1M to 2M within the same layer. The high level of lattice disordering of 2M illite suggested an early stage of 1M to 2M transformation. Thus, the diagenetic alteration of both apatite and illite crystals within the bone matrix may have increased its overall density, as the preferred orientation of apatite crystals from moderate to strong degrees was evident despite the poor preservation of osteohistological features. The combined effects of rapid burial, formation of a boundary region, and diagenesis of illite and apatite within the bone matrix may have contributed to the rib bone preservation.

## Introduction

The Boseong fossil site is a rich Upper Cretaceous fossil site from South Korea based on the discovery of abundant dinosaur eggs and egg clutches [1–3], post-cranial skeletal remains of the small basal ornithopod *Koreanosaurus boseongenesis* [4], and partial skeletal remains of the large anguimorph lizard *Asprosaurus bibongriensis* [5]. While it has been presumed that rapid burial took a key role in preserving the fossils from the local region based on large-scale depositional features [2], deeper insights in specific factors that may have contributed to bone preservation has not been thoroughly explored. Analytical studies on both skeletal fossil material and the hosting geological matrix provide insights into the interaction and relationship of composing elements [6,7]. Chemical analysis on a microscopic scale of fossil bone allows further evaluation of elements that have native and/or external origins [7–12]. Although the general nanostructure of fossilized bone can be investigated through novel X-ray techniques [13], transmission electron microscopy (TEM) analysis directly provides information on the morphology, arrangement, and chemistry of fossil apatite nanocrystals [9,14–18]. Such data are crucial for understanding how these apatitic crystalline phases have originated and also how the bone structure was maintained during fossilization. It should be noted that TEM data is based on an extremely small and localized scale, and such shortcomings of TEM investigation can be mitigated through correlative microscopy [17–19]. We have previously investigated structural and chemical features at micro- to nanoscale of a dorsal rib portion from *Koreanosaurus* [17] obtained from the holotype specimen (referred to as KDRC-BB2: Korea Dinosaur Research Center-Boseong Bibong 2) which consist mainly the “torso” region discovered in an articulated state in a large mudstone block that may have originated directly from the main outcrop of Site 5 [4]. In this study, the distal region of a fully preserved seventh left dorsal rib bone was obtained from KDRC-BB2, and we have specifically selected it based on the following reasons; i) information on exact original position, ii) simple morphology, and iii) intact hosting mudstone (S1 Fig).

Here, through correlative microscopy techniques, we aimed to investigate the microstructure and chemistry of key constituting phases of the fossil rib bone, hosting mudstone, and the boundary in-between. We evaluated the distribution and interaction of the key phases between these regions with focus on unraveling features involved in diagenesis and bone preservation. The frequent occurrence of “platy phases” within the rib bone matrix from our initial study was a compelling feature [17], and we intended to fully reveal the identity and origin of these phases and their presumed role in bone preservation. These phases may have affected the arrangement of apatite crystals from the rib bone, and without TEM investigation, such assumptions cannot be thoroughly assessed. Although our research sample represents only a small fraction of the entire skeletal fossil and fossil site, the preservation of the brittle and highly porous dorsal rib bones of *Koreanosaurus* was intriguing. We also considered it as an ideal research material without inflicting considerable damage to the holotype specimen. Due to the small size and fractured state of the rib bones, correlating microstructural and nanostructural preservation of osteohistological features were mainly performed on the larger and more intact femoral bones from the paratype specimens [4,18].

Correlative microscopy is a technique for performing progressive structure and chemical analyses on specimens from macro- to nanoscale and involves the use of optical and electron microscopes. The key importance of this technique is that a specific region of interest observed from the optical thin section should be reexamined in detail by electron microscopes in higher resolution. A crucial step in correlative microscopy is the use of proper and effective sample preparation methods for the corresponding analytical procedures. Typically, two types of samples—optical thin sections and nanopowders—are prepared directly from the bulk specimen. As conventional powder preparation methods result in an inevitable loss of spatial information, in this study we employed an ultrasonic drilling device (S2A Fig) which allowed us to acquire very small amounts of powders (in ng range) from predetermined specific regions either from the bulk specimen or optical thin section. We also designed an ultrasonic spraying device (S2B Fig) that is capable of dispersing powder samples on a TEM grid via a masking tool. This device is derived from a previously developed multi-sample loading device by our research team [20], which can load up to four samples on a single TEM grid using more conventional methods. We also utilized half-masking techniques on TEM grids by coating Au nanoparticles as a standard on half of the grid. For acquiring TEM samples from optical thin sections, we employed focused ion beam (FIB) milling. The biggest advantages are its precision and capability in preparing TEM samples from various directions [21]. The following methods were used in this study. 1) Initially, we used a stereoscopic zoom microscope to observe the bulk sample before carrying out specific preparation procedures. We also used the microscope to evaluate the overall sample quality after preparation using reflected light. 2) We used a polarizing optical microscope to identify mineral constituents of the hosting mudstone, and to discern the microstructural features of the rib bone. 3) We carried out initial phase identification by X-ray diffraction (XRD) analysis on both the optical thin sections and powder samples. 4) An electron probe microanalyzer (EPMA) equipped with wavelength dispersive spectroscopy (WDS) was primarily used for chemical mapping of optical thin sections to characterize the distribution of distinctive and common elements from the rib bone, hosting mudstone, and boundary region. 5) Scanning electron microscopy (SEM) imaging and chemical analysis with energy dispersive spectroscopy (EDS) was performed for investigating microstructures and elemental distributions from both thin sections and powder samples in general, and for characterizing the constituting phases. 6) We used TEM as the main technique for evaluating the specific structure and chemistry of the identified phases. For electron crystallographic analysis, we obtained and analyzed selected area electron diffraction (SAED) patterns and high-resolution TEM (HRTEM) images along with corresponding fast Fourier transform (FFT) data. We mainly carried out TEM–EDS analysis to identify the distribution of specific clay phases from the FIB-milled samples. All microscopes were equipped with charge-coupled device (CCD) cameras, and we captured and stored the micrographs directly using the respective software of each CCD camera.

## Materials and methods

### Research materials and depositional features of the Boseong fossil site

The fossil bearing Seonso Formation and underlying Seonso Conglomerate (see table in S1 Table for stratigraphic details) consists of clastic conglomerates, greenish gray to variegated sandy sandstones, alternating sandstones and mudstones, reddish to purple mudstones, and an abundance of features indicating the presence of rich carbonate content such as calcareous nodules, calclithite, and calcite-filled desiccation cracks [2–4,22]. Isotopic datings of ^40^Ar-^39^Ar in sanidine from Pilbong tuff and lapilli tuff located directly above and below these fossiliferous sediments, respectively, resulted in an estimated age of approximately 81 Ma, and the insignificant age differences between the two tuff layers implies rapid deposition of the fossiliferous sediments [3]. The upwards-fining units that frequently occur within the Boseong fossil site indicate formation from alluvial environments [2]. River floodplains may have been the likely source of rapid deposition, and the presence of calcic paleosols and burrows in overbank mudstones specify that the dominant process was sheetflooding [2]. Calcic paleosols may have also taken a contributing role in fossil preservation after burial [2,23], and its development along with vertic paleosols from the deposit suggest that flooding was perhaps not overly frequent [2].

We selected the distal portion of the seventh left dorsal rib bone of *Koreanosaurus* from the holotype specimen KDRC-BB2. KDRC-BB2 was discovered in Site 5 among the five dinosaur fossil egg sites located along the coastal areas of Bibong village, Boseong County [4]. Excavation, relocation, and preparation of the specimens were carried out by the Korea Dinosaur Research Center (KDRC) research team at Chonnam National University, Gwangju, South Korea. All skeletal specimens of *Koreanosaurus* are currently housed at KDRC and are on public display. For direct access of the *Koreanosaurus* specimens, prior contact with the KDRC director is required. Specific permits for the research material were not necessary for this study.

Although most of the skeletal elements of KDRC-BB2 were thoroughly prepared, due to the brittle nature of the dorsal rib bones, the surrounding mudstone was not completely removed, thus allowing us to obtain rib fossil specimens with the hosting mudstone intact. The acquired rib bone sample was around 10 mm in width, 46 mm in length, and it progressively flattened dorsoventrally towards the terminal end (S1 Fig).

### Optical microscopy (OM)

The obtained portion of the seventh dorsal rib bone was embedded in polyester resin (Epo-fix embedding kit, Electron Microscopy Sciences) and after curing for over 24 hours in room temperature, we serially sectioned the specimen using a diamond saw (Precision Saw, Buhler) and a diamond wire saw (Model 3242, Well). After polishing and mounting one side of the section on a slide glass, we ground and polished the other side until it reached the desired sample thickness of about 40 μm–70 μm. Osteohistological features from the bone region were best observed from a thickness of about 40 μm–50 μm. For sample quality assessment and an overall view of the microscopic features, we initially observed the optical thin sections through a stereoscopic zoom microscope (SMZ 1500, Nikon). A polarizing microscope (Eclipse E600 Pol, Nikon) was subsequently used for more thorough observations. These steps were repeated for every prepared optical thin section until we found the most ideal section which had even thickness in all regions (bone, boundary, and mudstone) for further analysis.

### XRD

Initial measurements were made from optical thin sections and obtained powders from the hosting mudstone with a powder XRD instrument (D8 Advance, Bruker). We performed subsequent analysis for specific peak identification using a high-resolution XRD instrument (D8 Discover, Bruker) with the following parameters: a 2θ range of 5° to 100°, in steps of 0.02°, with a step duration of 76.8 s for optical thin sections and 2 s for powders. Peak assignments were made automatically and manually with the software DIFFRAC.EVA Ver. 3 (Bruker). For the finalized data, we analyzed identical samples with an X’Pert-PRO MRD (PANalytical) with 2θ ranges of 5° to 90°, a step of 0.013°, and a step duration of 30 s. For a more thorough analysis of the clay phases, we prepared separate powders from the rib bone and measured them with a 2θ range of 5° to 30°, a step of 0.013°, and a step duration of 300 s.

### EPMA and WDS

The main optical thin section was coated with carbon at a coating thickness of approximately 20 nm for EPMA and SEM analyses. We used an EPMA instrument (Model 1610, Schmadzu) with a tungsten gun operating at 15 kV–30 kV (20 kV mainly used) with a maximum current of 100 μA and equipped with four WDS detectors with a detection limit of 100 ppm. Spot (size 10~20 μm) scan analysis was initially conducted to determine specific elements that should be included for mapping. The following specific elements were selected for elemental distribution mapping using the built-in software program (Schmadzu) for the main optical thin section: Na, Mg, Al, Si, P, K, Ca, Ti, and Fe.

### SEM and EDS

Initial viewings and analysis was done through an environmental scanning electron microscope (LEO 1455VP, Carl Zeiss) operating at 20 kV, equipped with an EDS detector (Vantage LN_2_ type). To obtain higher resolution SEM images we used a field emission SEM (FE-SEM, Merlin, Carl Zeiss) operating at 0.02 kV to 30 kV. Chemical analysis was performed with an EDS system (XFlash 6160; a software of Espirit 1.9.4, Bruker) which was attached to the FE-SEM. We conducted EDS mapping on the following elements: F, Na, Mg, Al, Si, P, K, Ca, and Fe.

### TEM

#### TEM sample preparation

TEM samples were prepared directly from the optical thin section with a FIB miller (Quanta 3D FEG, FEI) equipped with a field emission gun operating at 0.5 kV to 30 kV. We selected and marked specific regions after EDS analysis (Apollo X with an EDAX software, Genesis Spectrum version 6.41). We separately prepared cross and plane samples and loaded them onto specialized TEM grids (Lift-Out Grids, Omniprobe) suitable for the FIB-milled samples. The thickness of the FIB-milled samples was about 80 nm. For the FIB milling process, we used the built-in software programs xT microscope Control, xT microscope Server, and FEI User Management (FEI). For obtaining powder samples, we employed ultrasonic drilling from predetermined regions from the bulk sample and optical thin sections. We collected powdered samples by dispensing and absorbing ethanol via a pipette and then placing the solution in an ultrasonic sprayer to disperse the powders onto a TEM grid. Half-masked TEM grids coated with Au were also used to calibrate the SAED patterns.

#### TEM Instruments

A field emission TEM (FE-TEM, JEM-2100F, JEOL, operating at 200 kV) and a monochromated field emission, energy filtering TEM (FE-EFTEM, Libra MC, Carl Zeiss, operating at 60 kV–200 kV) were employed to obtain the TEM micrographs, SAED patterns, HRTEM images, and EDS data. The specifications for instruments used for SAED and EDS analysis are: (i) JEM-2100F: side CCD (sensor size: 2048 x 2048, pixel size: 14 μm) for SAED (beam stopper used); EDS detector (EX-24065JGT, JEOL) and software (Analysis station version 3.63.01, JEOL); (ii) Libra MC: side CCD (sensor size: 2048 x 2048, pixel size: 7.4 μm) for SAED (beam stopper not used); EDS detector (X-Max 80T, Oxford) and software (Aztec version v2.2 SP2, Oxford). TEM micrograph processing, measurements of the SAED patterns and HRTEM/FFT images were done with the software Digital Micrograph version 1.72.53 (Gatan).

## Results

### OM

**Fig 1:**
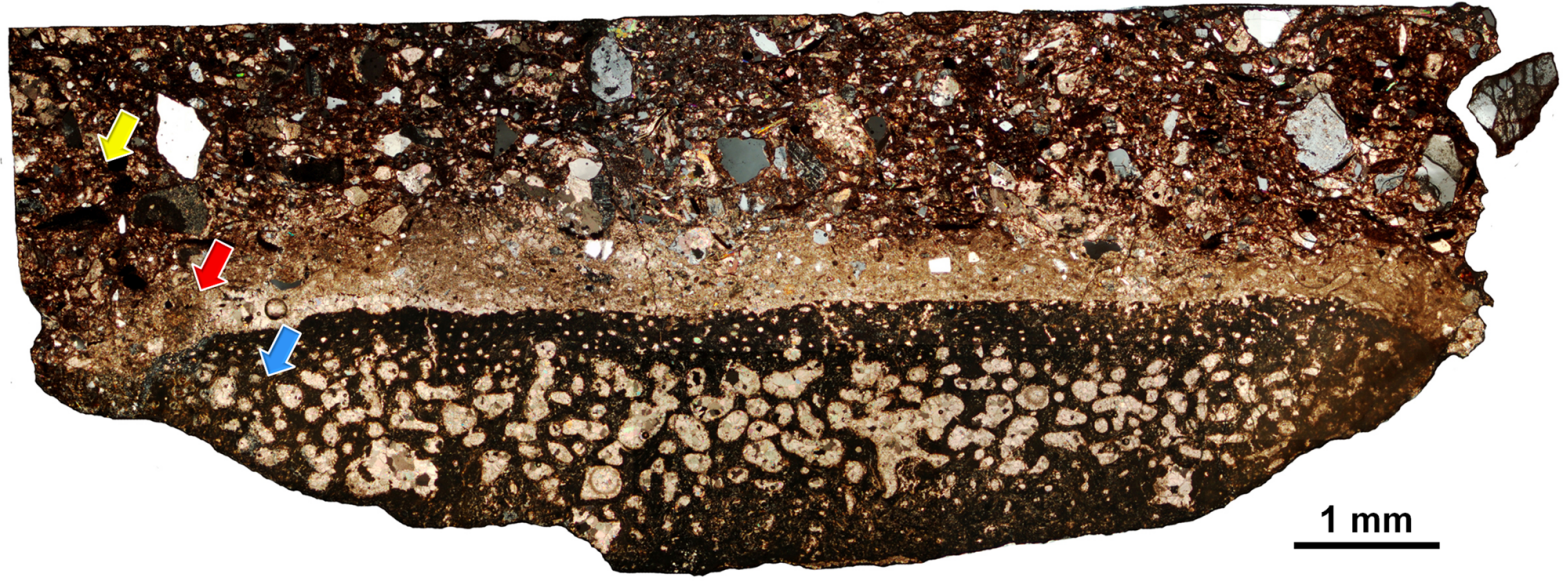
Composite cross-polarized optical micrograph of the main thin section (x40). The section was divided into three regions–hosting mudstone (yellow arrow), boundary (red arrow), and rib bone (blue arrow). Clusters of calcite microcrystals can be directly observed in all regions. The mudstone and boundary region primarily contains detrital clasts of quartz and feldspars. Due to the compressed nature of the bone matrix, specific osteohistological features were not discernible from the rib bone besides the vascularization pattern.

Fig 1 shows the overall optical micrograph of the main thin section which we used in our correlative OM-XRD-EM study. This section can be divided into three distinct regions; (i) hosting mudstone, (ii) boundary, and (iii) rib bone. The major constituents of the hosting mudstone appear to be cryptocrystalline clay minerals. Larger clasts have predominantly angular morphology, which indicates that they have primarily detrital origins. Quartz was the most notable in the clasts in various sizes ranging from smaller than 10 μm to larger than 300 μm. Calcite was also prominent and these clasts were formed by clusters of inequigranular microcrystals of various orientations. Individual calcite microcrystals were a mixture of anhedral and nearly subhedral crystals. In every thin section, we observed a large distribution of smaller calcite clasts and quartz clasts interspaced within the mudstone matrix. Feldspars were relatively harder to discern and required chemical analysis for clarification. We identified albite, sanidine, and andesine. The sanidine and andesine clasts were relatively large (200–400 μm) and sparsely distributed, but most of the albite clasts were smaller than 50 μm and very abundant.

An intriguing feature of the specimen was the presence of a middle boundary region which had an overall thickness of about 500 μm. The clasts within this region overlapped with the mudstone region, but there were far fewer visible clasts and their sizes were generally smaller. Quartz clasts were the most noticeable and displayed anhedral to subhedral morphologies. Based on the high-magnification images, the boundary matrix appeared to be primarily composed of very fine-grained calcite, and lacked distinct consecutive layers. Due to the presence of clay minerals interspaced within the calcite matrix, the overall appearance differed from the calcite clasts of the mudstone region and from those filling the larger pores of the rib bone region. Certain regions within the boundary that lack clay content show fully crystallized clusters of calcite microcrystals with individual grains around 10 μm in diameter, which are almost identical to those found in the mudstone and bone pore regions (S3 Fig).

Apart from identifying vascularization patterns, discerning osteohistological features of the rib bone proved to be difficult due to the compressed state of the bone. The vascularization patterns displayed a trend of sharply increasing in size and shape irregularity from the outer region towards the center, which is a feature consistent with our previous investigation [17]. Large cracks and visible pores of the rib bone were occupied by calcite microcrystals of various orientations. To discern specific clay phases and their distributions, we performed EM imaging and chemical analysis.

### XRD

**Fig 2:**
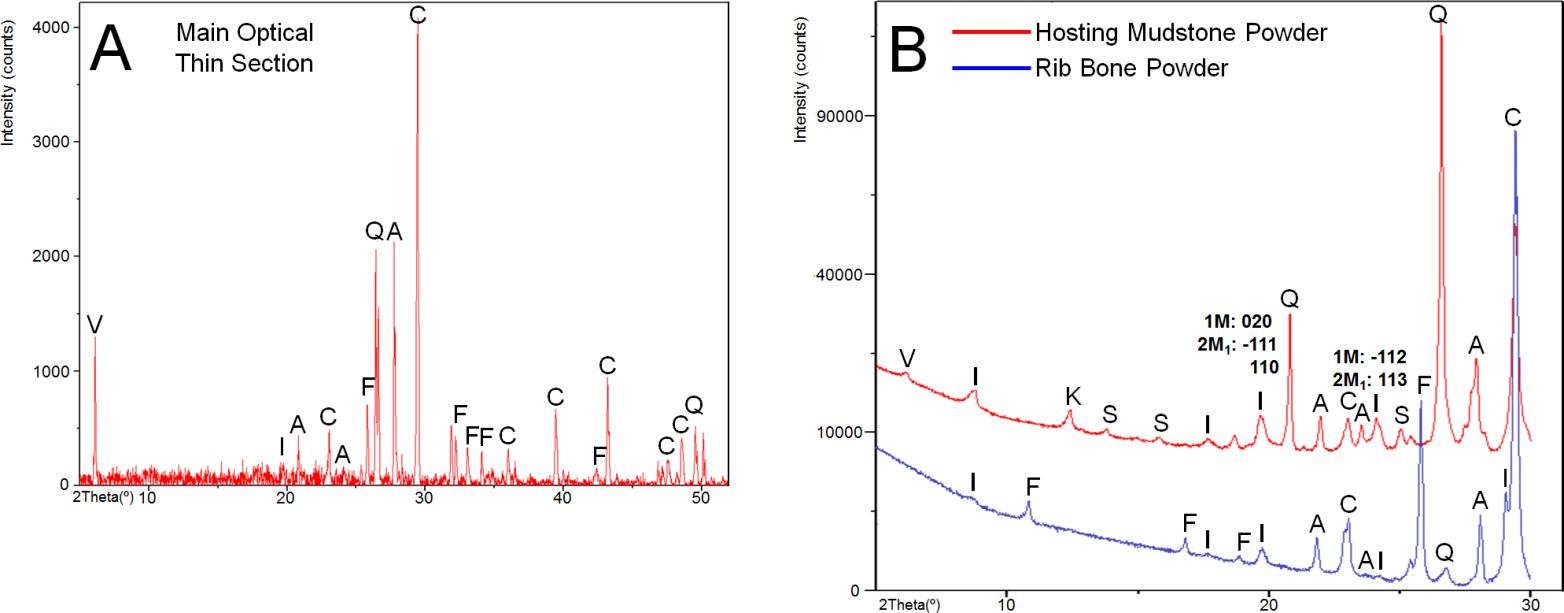
XRD analysis. (A) Main optical thin section (noise background removed). Major phases were calcite, quartz, albite, and fluorapatite. Vermiculite and illite were distinctly identified clay phases. (B) Hosting mudstone powder and rib bone powder focused on identifying clay phases. Clay phases besides illite were not detected from the rib bone sample. The prominent illite peaks at 20° range and the peaks in 24° range in the mudstone sample indicate that both 1M and 2M_1_ illite polytypes are present. A = albite, C = calcite, F = fluorapatite, I = illite, K = kaolinite, Q = quartz, S = sanidine, V = vermiculite.

As presented in Fig 2, we used XRD to analyze the main optical thin section and the powder samples obtained from the hosting mudstone matrix and the rib bone. From the main optical thin section (Fig 2A), we identified the major phases as calcite, quartz, albite, and fluorapatite. The illite peaks were weak or negligible mostly due to signals from the embedding resin, but we identified a sharp vermiculite {002} peak, which is an unusual feature (and which appeared consistently in the data even by different XRD instruments), contrast to other optical thin sections which had relatively weak vermiculite peaks. We are uncertain why there is such a strong vermiculite peak exclusively in the main optical thin section.

From powder samples we identified illite, kaolinite, and vermiculite in the mudstone, and only illite in the bone (Fig 2B). The strong illite peaks in the 20° and 24° ranges in the mudstone sample indicate that both 1M and 2M_1_ illite polytypes are likely present [24–27]. The strong presence of the illite peak in the 20° range from the bone sample suggests that 2M_1_ illite might be common, and we have carried out EM investigation to clarify which specific type of illite is mainly occupying the small pores of the bone matrix. Fluorapatite peaks appeared only in the bone sample, while calcite, albite, and quartz were present in both the mudstone and bone samples.

### EPMA

**Fig 3:**
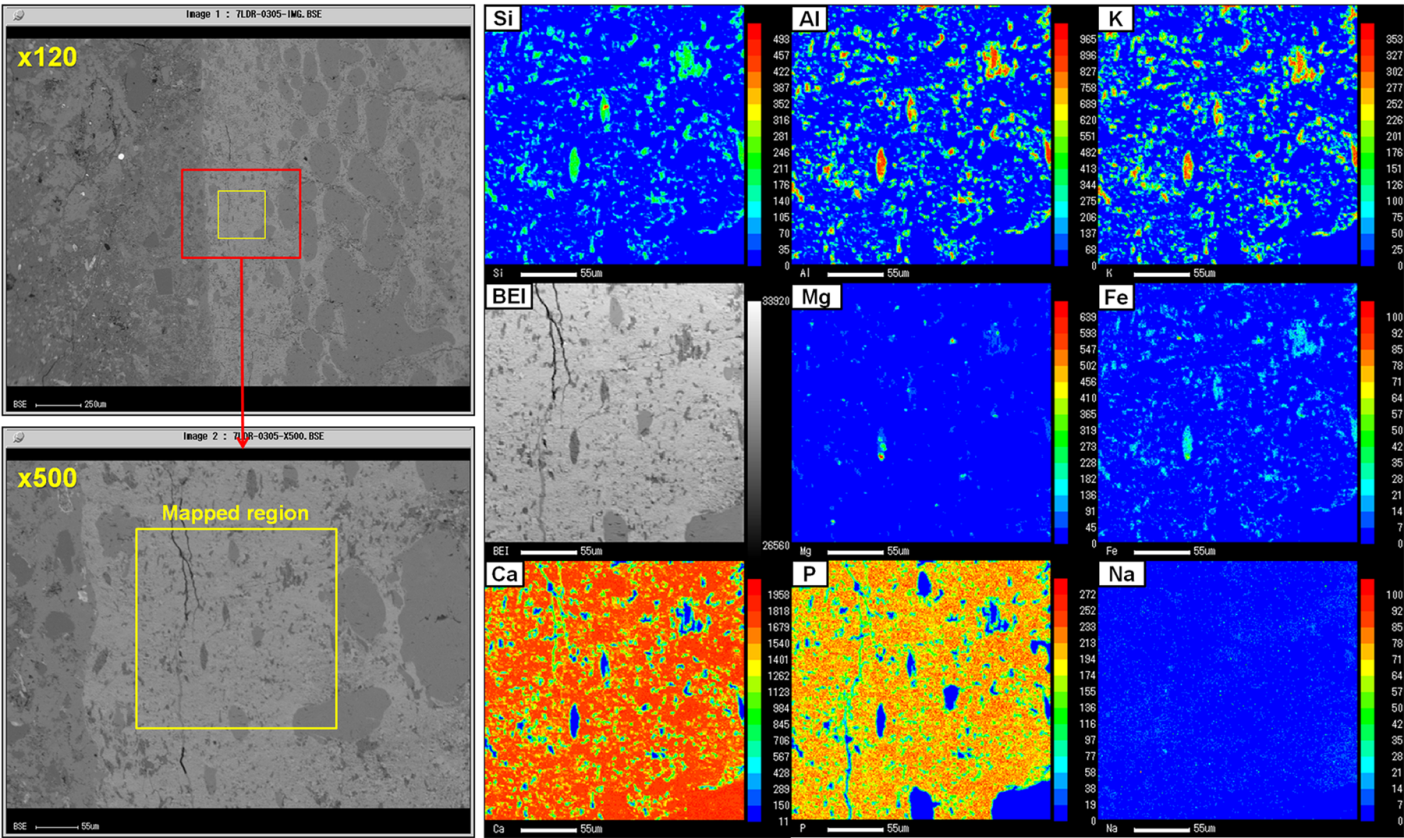
EPMA mapping of the rib bone adjacent to the boundary. Due to calcite and calcium content in the bone region, the distribution and concentration of Ca is very high. The most striking feature is that miniscule pores within the bone matrix and some of the smaller vascular channels are occupied with illite (Al, Si, K, Mg, Fe) instead of calcite. BEI = backscattered electron image.

EPMA element distribution mapping was carried out on the main optical thin section. The concentration of specific elements is indicated by count bars placed on the right side of each individual element map (Fig 3, S4 Fig).

(i) Hosting mudstone: We selected a specific region with the least number of visible clasts. Si, Al, K, and Mg were the most widely distributed overlapping elements, further indicating illite as the main phase of the mudstone matrix. Fe also had an overlapping distribution with the above main elements, albeit in a relatively lower amount. We found a few areas of spiked Mg distribution that were lacking in K but overlapping with Al and Si, which revealed a distribution of vermiculite. A sparse distribution of small quartz, calcite, and albite clasts were identified, which was a common feature throughout the mudstone matrix.

(ii) Boundary region (S4 Fig): Several areas were mapped within the region, which revealed a prominent distribution of Ca. The Ca concentration increases near the rib bone due to the presence of clusters of microcrystalline calcite (S3 Fig), whereas the Ca distribution sharply decreases toward the hosting mudstone region. One of the features we expected to observe in the region was a wide distribution of P, but our results indicate that P has been retained almost exclusively in the rib bone matrix. An overlapping distribution of Si, Al, K, Mg, and Fe is present in the boundary region, and although the overall concentration of these elements is relatively low compared to the hosting mudstone, it is widespread throughout the region. The sparse, overlapping Al, Si, and Mg and lack of K signified the presence of vermiculite. Most of the clasts were identified as quartz. Other minor clasts included small amounts of albite (clasts mostly smaller than 30 μm) and magnetite. A significant feature of this region was the complete separation of Ca from the clay elements, despite their appearance of being intermixed, thus excluding the possibility of the formation and presence of Ca-Si compounds.

(iii) Rib bone (Fig 3): Ca and P were found to be the main elements of the matrix, and the concentration of Ca was extremely high relative to other mapped elements. P distribution was virtually exclusive in the matrix, signifying the presence of apatite. Overlapping distributions of Al, Si, and K (illite) were evident, and were mostly concentrated in miniscule pores and gaps (smaller than 20 μm) within the bone matrix. A few of the smaller vascular channels were also occupied by illite. Although Mg and Fe were present in the matrix and showed overlapping distributions with Al, Si, and K, their concentrations were low, especially Mg. The distribution of illite within the bone matrix (excluding vascular channels) showed preferential occupation of pore spaces based on its size and shape differences with calcite.

### SEM

**Fig 4:**
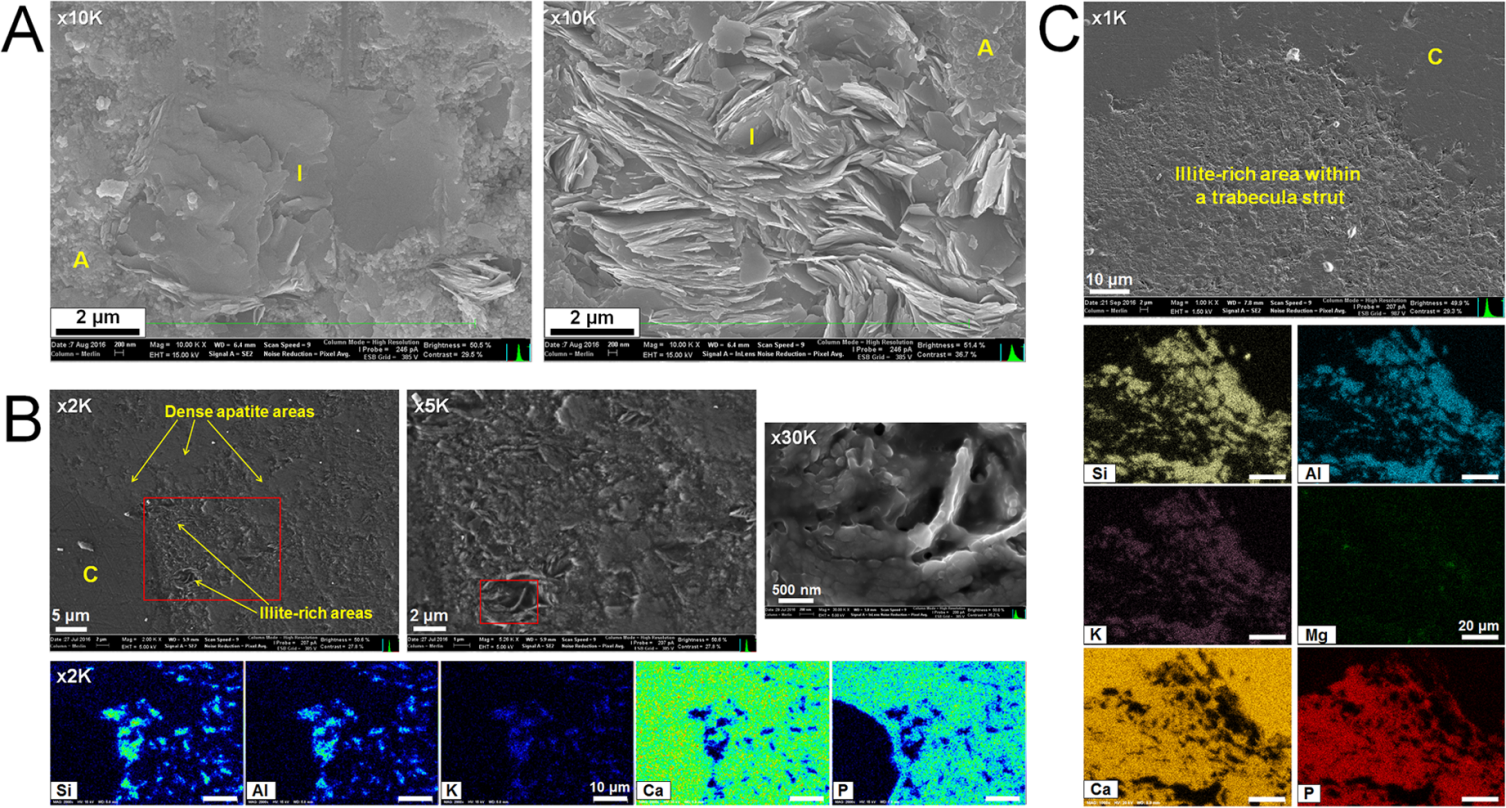
SEM analysis of illite within the rib bone matrix. (A) Illite crystals occupying small pores in varying directions. (B) Illite within the compact bone region and the corresponding EDS map. (C) Illite within the cancellous bone region and corresponding EDS map. There was a wider distribution of highly concentrated illite in the trabeculae struts. A = apatite, C = calcite, I = illite.

SEM imaging was primarily used to evaluate the morphology and placement of illite within the rib bone matrix. We found illite to be generally distributed in various directions within minuscule pores and gaps (Fig 4A), and also to have various orientations when occupying small vascular channels (S5A Fig). The platy morphology of illite was a prevalent feature and we detected no other morphology types via imaging.

As shown in S5B Fig, we used EDS mapping to evaluate the relative distribution of key elements. The distribution of the main elemental phases such as Ca (calcite), Al, Si, and K (illite) can be seen from every region. The boundary region is especially rich in Ca, Si and Al, and while Si and Al generally display an overlapping distribution excluding quartz clasts, Ca shows a distinct non-overlapping distribution with these elements. Separate mapping data of the rib bone region show an overlapping distribution of Ca, F, and P, indicating that the bone matrix is mainly constituted of fluorapatite.

High-magnification EDS mapping was performed on the bone matrix primarily to evaluate the distribution of illite (Fig 4B and 4C). We found a rich and wide distribution of illite within the bone matrix. While the overall bone matrix porosity of both the compact and cancellous bone was very high, the cancellous bone region had wider illite distribution (Fig 4C). High-magnification EDS mapping of the boundary region revealed that while illite was the main clay phase, vermiculite was also clearly present, albeit sparsely, and was usually located between illite. The concentrated distribution of vermiculite was relatively high in areas where the hosting mudstone and boundary meets, which can be seen in the Mg map of S5B Fig.

### TEM

An optical thin section directly facing the main thin section was selected for preparing the TEM samples, and FIB milling was utilized to obtain TEM samples from the cross and plane directions (sampling positions shown in S6 Fig). Powder samples were also prepared from both the bulk sample and optical thin sections with an ultrasonic drill. We mainly focused on identifying the clay phases and investigating their microstructures and chemistry. We also investigated the arrangement of the apatite phases within the rib bone matrix by SAED pattern analysis [18].

#### Clay structure and chemistry analyses

**Fig 5:**
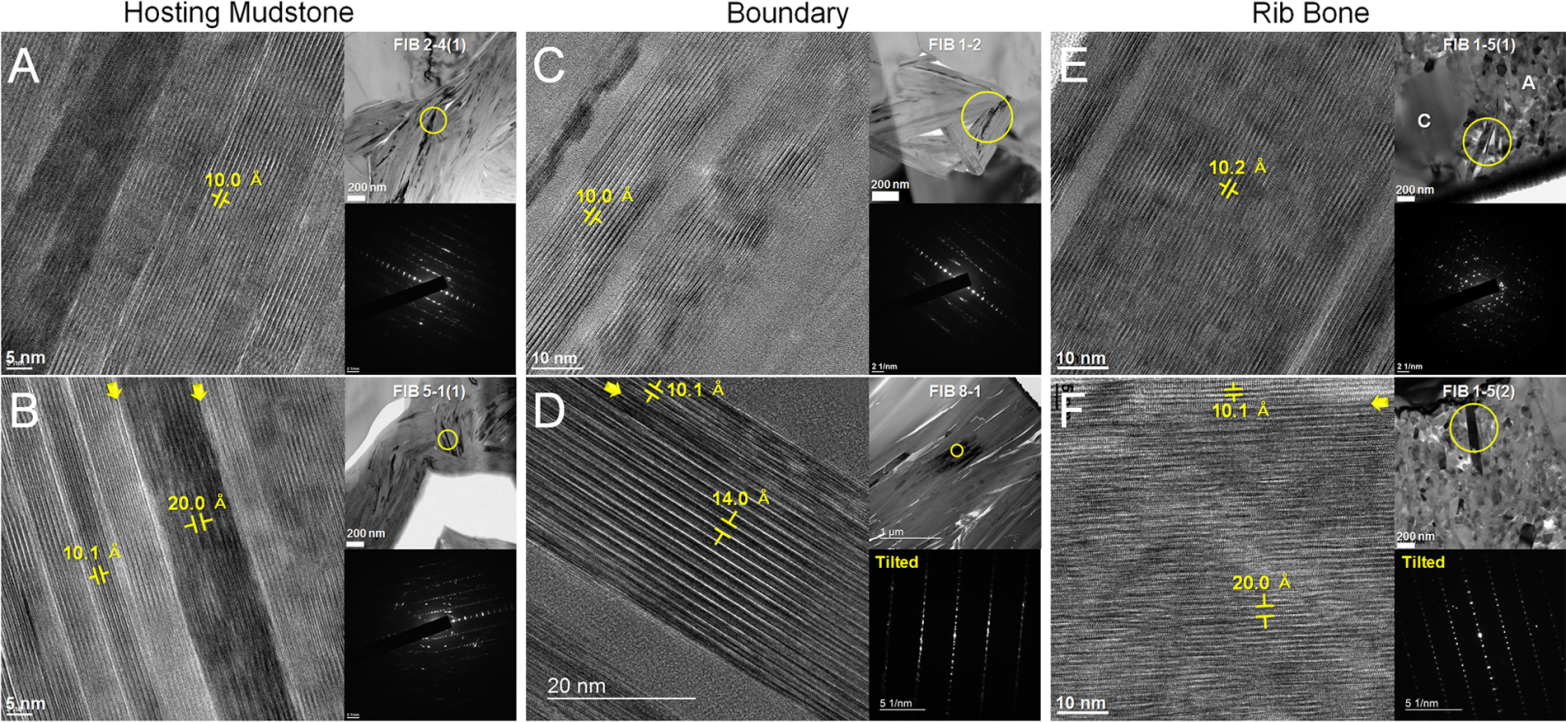
**HRTEM images and SAED patterns from the designated areas (yellow circled) in TEM micrographs of clay**; mudstone ((A) and (B)); boundary ((C) and (D)); bone ((E) and (F)) regions. The parallel boundaries between 1M and 2M illites are arrowed in (B) and (F). The parallel boundary between illite and vermiculite is arrowed in (D). Note the highly disordered {00l} 2M lattices in (B) and (F), especially in (F). A = apatite, C = calcite.

We obtained more than 100 TEM lattice images of clay phases from 23 FIB-milled samples along with the TEM-EDS data (The overall data are provided in the table in S2 Table). Table 1 shows the {00l} interplanar spacings and chemical data of the typical clay phases of each region. Because of the severe electron beam damage of TEM samples, TEM-EDS analysis was performed mainly through area scan analysis, followed by area mapping in scanning transmission electron microscopy (STEM) mode instead of point analysis. A majority of the analyzed clay phases had interplanar spacing of around 10 Å and chemical compositions of Al, Si, K, with minor amounts of Mg and Fe, which indicated that the 1M-type illite was prevalent in the TEM samples from all three regions. With an interplanar spacing of around 20 Å, we occasionally observed the 2M-type illite together with the 10 Å illite. Certain clay phases had interplanar spacings exceeding 14 Å and were particularly rich in Mg, and along with the XRD, EPMA, and SEM–EDS data, we identified these clay phases as vermiculite [25,28]. Vermiculite was only observed in the boundary and mudstone regions through TEM analysis. Fig 5 shows lattice images of the representative clay phases from each region along with their respective SAED patterns (additional data in S7 Fig). As shown in Fig 5A and 5E, the {00l} lattice fringes of the illite phase indicates that the 1M-type illite had low to moderate disorder mainly originating from the stacking disorder and lattice defects such as dislocations [29,30]. This disorder tended to be more prominent in the 1M-type illite of the bone region. The 2M-type illite showed a lattice arrangement that was parallel to that of the 1M-type illite, but the {00l} lattices were disordered to a much higher degree (Fig 5B and 5F). Vermiculite also had a parallel lattice arrangement with the 1M-type illite, as shown in Fig 5D, and also had a low-to-moderate degree of lattice disorder (Fig 5D, S7A and S7D Fig).

**Table 1:** The interplanar spacings, and chemistry of representative clay phases from each region.

Region	Mudstone				Boundary				Rib Bone			
Sample #	2-4(1)	2-4(2)^a^	5-1(1)	5-1(2)	1–1	1–2	1–3	8–1	1-5(1)	1-5(2)	1-5(3)	2–5
**Interplanar Spacing (Å)**	10.0	10.0	10.1	10.3	14.6*	10.0	10.0	10.1	10.2	10.1	10.0	-
	-	+14.6*	+20.0	+20.0	-	-	-	+14.0*	-	+20.0	+20.0	-
**SiO**_**2**_	43.92	40.10	47.28	41.75	29.64	44.88	40.85	45.34	50.30	54.44	43.95	41.03
**Al**_**2**_**O**_**3**_	42.00	37.71	37.73	43.17	27.46	43.53	37.48	27.76	36.66	35.15	39.67	40.93
**K**_**2**_**O**	14.08	4.52	8.90	8.24	0	5.98	7.42	4.68	7.27	5.24	10.82	11.46
**MgO**	0	12.93	2.92	3.72	34.69	2.15	8.87	17.27	2.23	2.17	2.77	3.05
**FeO**	0	4.74	3.32	3.12	8.21	3.46	5.38	4.95	3.54	3.00	2.80	3.53
**Total**	100	100	100	100	100	100	100	100	100	100	100	100
**Scan type**	Area	Area	Area	Area	Area	Area	Area	Map	Area	Area	Area	Map
**Corresponding Figures**	5A	S7A	5B	S7B	S7D	5C	S7C	5D	5E	5F	6	7

^a^ Interplanar spacing data obtained from HRTEM image and corresponding FFT data.

* Vermiculite.

Sample 2–5: Plane view.

**Fig 6:**
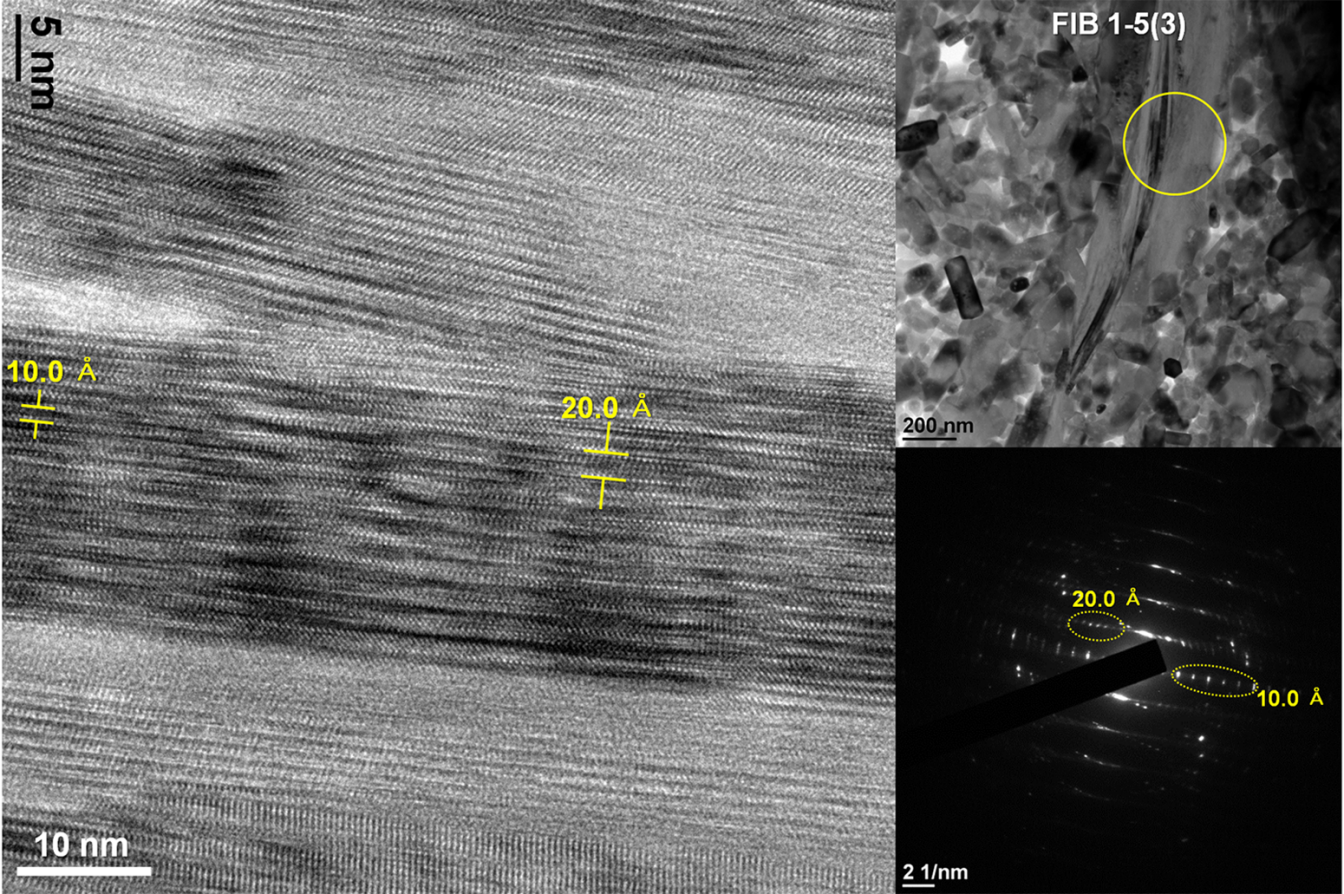
An area showing lateral variation of the 1M-type illite and the 2M-type illite within the same layer. The gradual transformation of the 1M to 2M illite in a lateral direction is apparent. Note the highly disordered {00l} lattices of 2M from the HRTEM image.

Even though the total numbers of observation points were quite small, the association of the 1M- and 2M-type illite was of our particular interest, as it may be an indication of their reaction or transformation mechanisms [31]. The boundaries between the IM- and 2M-type illites observed in the mudstone region were sharply defined in one side, but rather gradual in the other (arrowed in Fig 5B and S7B Fig). However, their boundaries observed in the bone region were distinctly gradual (arrowed in Fig 5F and S7F Fig). We also observed a direct lateral variation of the 1M-type illite to the 2M-type illite in the bone region as shown in Fig 6. The 1M-type illite appeared to be transforming gradually to the 2M-type illite in the lateral direction maintaining the overall layer arrangement. The {00l} lattices of the 2M-type illite were highly disordered as in case of the other TEM samples. No peculiar chemical data other than those of typical illite compositions were noticed from TEM-EDS analysis of these samples (refer to Table 1).

**Fig 7:**
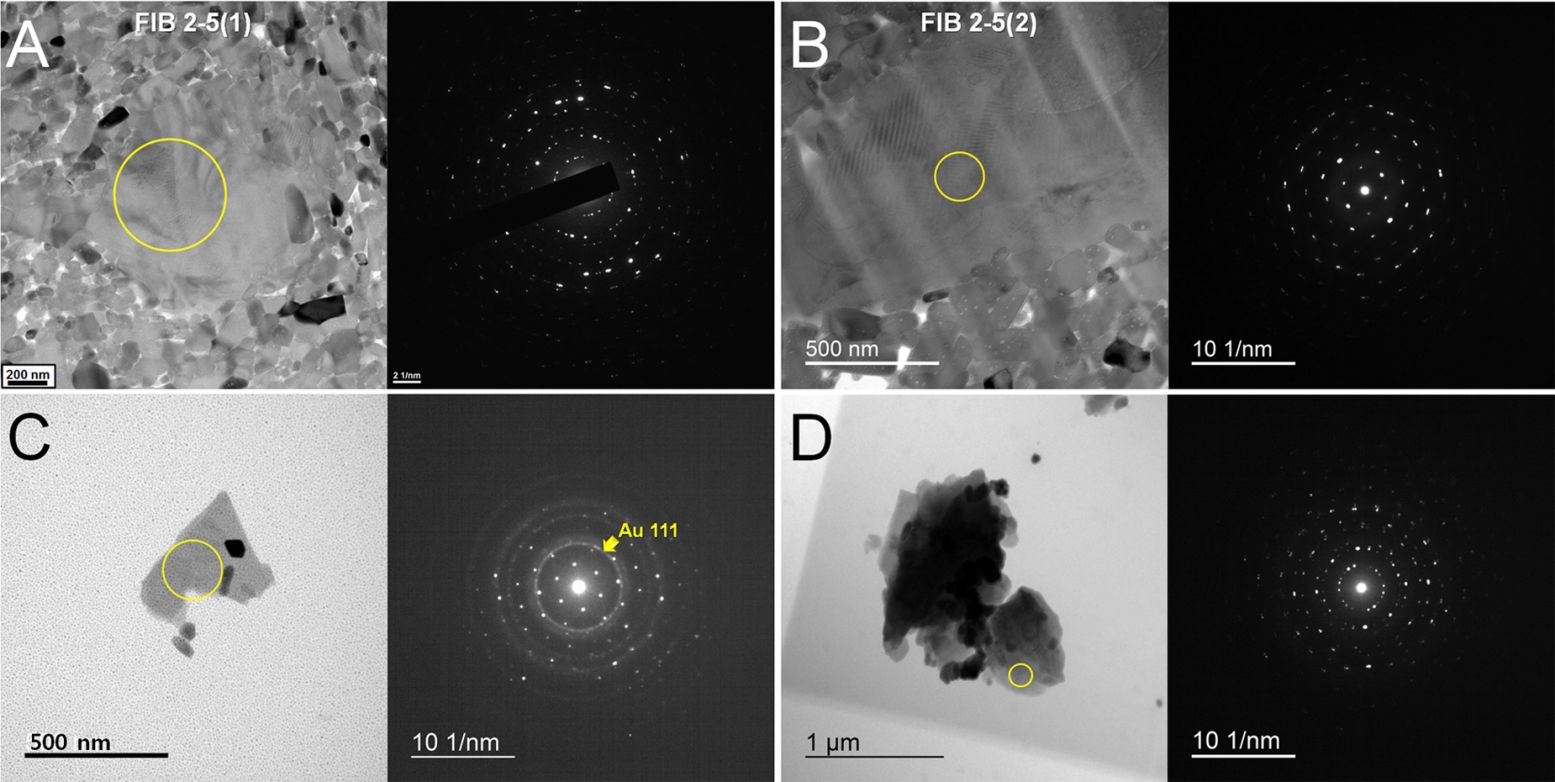
TEM micrographs and SAED patterns from the designated areas (yellow circles) of illite obtained from the rib bone region. ((A), (B)) The platy morphology of illite observed from a FIB-milled sample. SAED pattern indicates that illite “plates” are layered in rotation with one another. ((C), (D)) Illite “plates” obtained via ultrasonic drilling and spraying. (C) The tiny “specs” are Au nanoparticles used as the internal standard for d-space measurements. The smaller crystals are apatite. (D) SAED pattern indicates that illite “plates” are layered with some rotation with one another.

With respect to their platy nature, we studied illite in the bone region using two different TEM sample preparation methods; (i) FIB-milling of a vascular-channel-filled illite (plane view) and (ii) ultrasonic drilling of both the vascular-channel-filled illite and bone matrix illite. To obtain accurate d-spacing data, we loaded the powder samples onto a single TEM grid with its half area designated for the Au internal standard [20,32]. Fig 7 shows the obtained TEM micrographs and SAED patterns (refer to Table 1 for chemical data). The arrangement of the individual plate perpendicular to the {00l} plane demonstrates a mostly rotational relationship (Fig 7A and 7D), although sometimes with only small rotation angles (Fig 7B). Our analysis of the [1] SAED pattern with the Au standard rings (Fig 7C) yielded the following lattice data: d_020_ = 4.61Å, d_200_ = 2.57Å. The angle between d_020_ and d_200_ = 88.6°.

#### Apatite texture analysis

**Fig 8:**
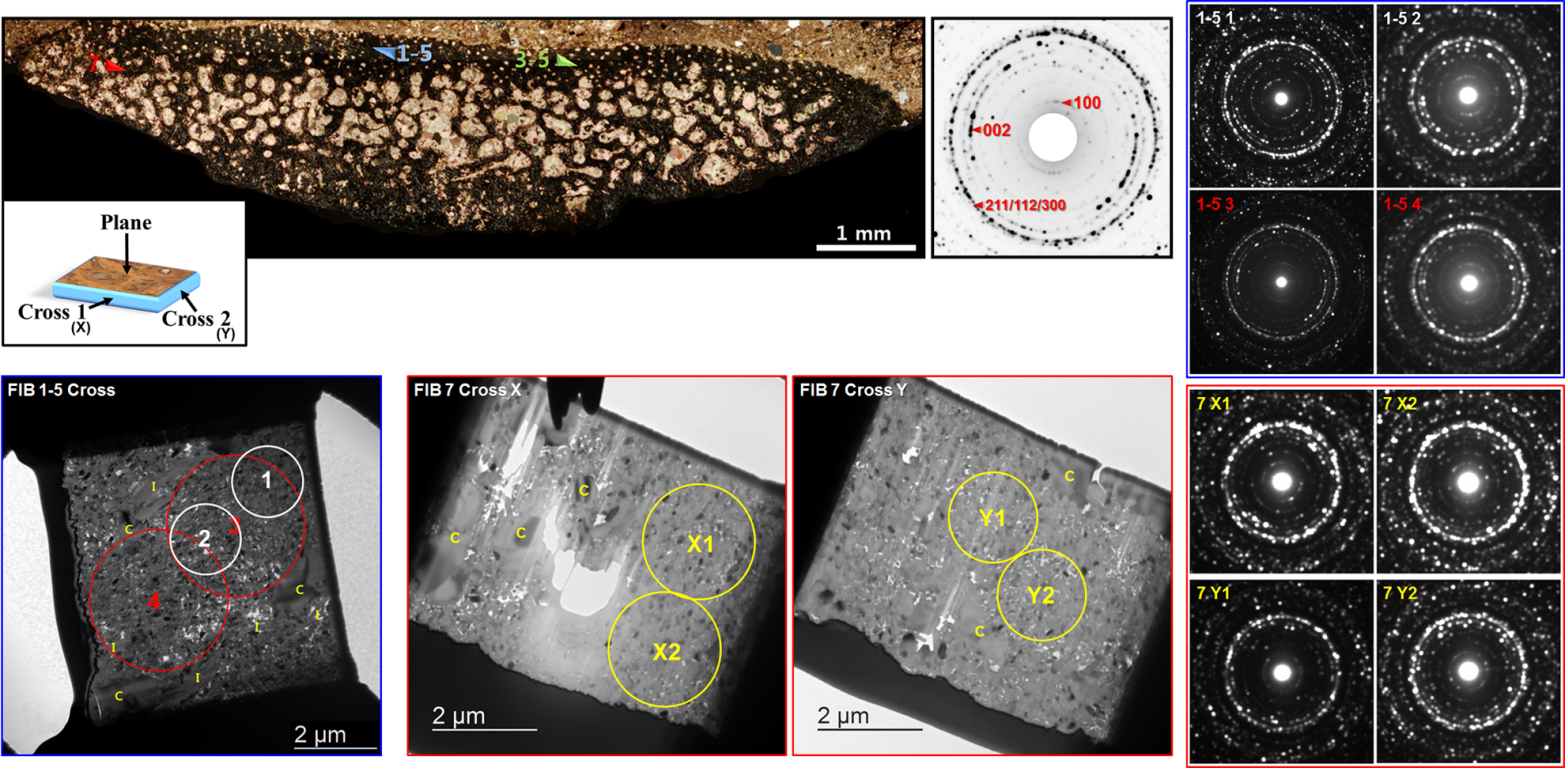
TEM micrographs and SAED patterns of apatite crystals from the selected areas (circles) of FIB 1–5 and FIB 7, obtained from the rib bone matrix. FIB 1–5 was obtained from an area with rich clay content, and all SAED patterns from areas with dense apatite population resulted in moderate to a slightly strong degree of preferred orientation. Both FIB 7 cross samples were milled from areas notably lacking in clay content. Besides cross X1, all SAED patterns lacked preferred orientations. C = calcite, I = illite.

Based on our chemical analysis, the apatite phase from the rib bone region was primarily fluorapatite. The crystals exhibited subhedral to nearly euhedral morphology, and the size range was a few tens of nm to over 200 nm based on the long-axis length. Most of the crystals were around 100 nm in length, consistent with prior values obtained from the fossils [17]. Some of the SAED patterns obtained from the cross-FIB-milled (parallel to the long axis of the rib bone) samples showed preferred orientations of apatite crystals in varying degrees. Initially, we selected FIB 1–5 and 3–5 based on having a sufficient amount of apatite crystals, the association of illite, and their orientations being perpendicular to each another. We milled FIB 7 to perform a comparative structural analysis with FIB 1–5 and 3–5, and obtained samples from a specific region that was notably lacking illite. Due to the highly porous nature of the rib bone matrix, regions with a very dense distribution of pure apatite were unobtainable. Fig 8 shows the specific sampled regions. We evaluated the degrees of preferred orientation mainly based on the length and intensity of “arc” rings of the {100} and {002} planes [18], which are in a perpendicular arrangement. The overlapping {211}, {112}, {300} rings have the highest intensity [33] and this feature was the main criteria for verifying that the SAED patterns were taken directly from apatite crystals.

The SAED patterns of the apatite crystals from FIB 1–5 all showed a moderate to slightly high degree of preferred orientation based on the length, shape and intensity of the “arcs” from the {002} diffraction rings [18]. By using different aperture sizes, we showed that preferred orientation was maintained by the areas with an overall dense apatite distribution within the sample. Although the osteohistological features were hindered, this provides direct evidence that the bone nanostructure is preserved to a certain degree at specific locations rich in illite. We prepared two cross-FIB-milled samples from FIB 7 along with a plane-milled (perpendicular to the long axis of the rib bone) sample and found all the SAED patterns to lack a preferred orientation of apatite crystals with the exception of cross X1, which showed a low degree of preferred orientation (Fig 8).

As shown in S8 Fig, all plane samples lacked a preferred orientation of apatite crystals, which implies that apatite crystals were arranged along the long axis of the rib bone. These results also show that obtaining proper electron diffraction patterns from apatite crystals is essential for evaluating bone nanostructure [18], as the distinctive ordering of apatite crystals are unclear based only on TEM micrographs and orientation mapping, although they do provide a direct view of the shape and size of the crystals.

## Discussion

The overall clast size, rounding, and sorting results from our samples indicate that the depositional energy was relatively low and the transport distance of the grains was presumably short. The reddish to purple hue of the hosting mudstone matrix is due to iron oxide minerals such as magnetite and ilmenite, and the presence of these minerals indicates the high amount of oxidation that occurred in the depositional environment [34].

### Phase distribution from the three regions

The identified phases and their distribution revealed through correlative microscopy are shown in Table 2. The key phases are illite, calcite and fluorapatite. Other notable phases are quartz, which was the most abundant clast; albite, also an abundant clast; magnetite, which has appeared in all regions albeit sparsely; and vermiculite, which was relatively more abundant in the boundary region.

**Table 2:** Identified phases and their distributions in each region of the samples studied.

Region Phase	Mudstone	Boundary	Rib Bone
Apatite (Fluorapatite)	Distribution: Only tiny specs of phosphorous were detected, thus apatite is virtually absent in the hosting mudstone.	Distribution: Detected only from a very small number of bone fragments in the region closely adjacent to the bone surface.	Distribution: Main phase of the bone matrix region.
			Appearance: Composed of tightly packed euhedral fluorapatite crystals ranging from 80~200 nm. Varying degrees of preferred orientation along the long axis of the bone
Calcite	Distribution: Widely distributed throughout the entire region as clusters of calcite microcrystals occupying the pore spaces.	Distribution: Widely distributed throughout the entire region and is the main constituent of the matrix.	Distribution: Widely distributed as clusters of calcite microcrystals filling pores and cracks in the bone.
	Appearance: Individual microcrystals usually around a few microns to 30 μm of varying orientations tightly packed in clusters mostly under 200 μm. Only a few clusters exceed 300 μm in width.	Appearance: Individual crystals of submicron to a few microns scale prominent throughout regions with rich clay content. A thin cluster of calcite microcrystals nearly 2 mm of length adjacent to the bone region is composed of microcrystals usually around a few microns to 30 μm.	Appearance: Individual microcrystals usually around a few microns to 30 μm tightly packed in clusters correlating to the size of the pores. Pores typically exceeding 40 μm are filled with calcite microcrystals.
Clays Illite	Distribution: Main constituent of the mudstone matrix.	Distribution: Widely distributed and secondary constituent of the matrix.	Distribution: Widely distributed in smaller pores and the matrix.
	Appearance: Exists as thin plates of varying orientations. Illite crystals occupy even extremely constrained spaces.	Appearance: Exists as thin plates of varying orientations. Usually wedged between larger calcite crystals.	Appearance: Exists as thin plates of varying orientations.
Vermiculite	Distribution: Minor clay phase throughout the matrix. Detected and identified through XRD analysis.	Distribution: Secondary clay phase, concentrated near the mudstone.	Not detected.
		Appearance: Exist as thin plates of varying orientations.	
Kaolinite	Distribution: Minor clay phase throughout the matrix. Detected and identified through XRD analysis.	Not detected.	Not detected.
Quartz	Distribution: Widely distributed throughout the entire region as clastic grains, and is the most abundant visible grain.	Distribution: Most widely distributed among visible detrital grains.	Distribution: Sparsely distributed throughout the matrix region (XRD), directly observed by EM.
	Appearance: Individual grains range from a few μm to over 300 μm in width and are in varying orientations. All grains are anhedral and grains exceeding 200 μm are sparse.	Appearance: Individual grains of varying orientations range from a few microns to over 200 μm in width although mostly under 50 μm. All grains are anhedral.	Appearance: Individual crystals with virtually round morphology in submicron scale. Usually around a few hundred nanometers.
Feldspars Albite	Distribution: Widely distributed throughout the region	Distribution: Common throughout the region, secondary clast phase.	Distribution: Sparse throughout the region (XRD), not directly observed by EM.
	Appearance: Anhedral grains usually around and under 50 μm in width.	Appearance: Anhedral grains usually around and under 30 μm in width.	Appearance: Grains around or under 10 μm.
Sanidine	Distribution: Moderately distributed throughout the region, secondary feldspar phase.	Not detected in the main sample, was not discernible through chemical mapping due to overlapping chemical elements with illite.	Not detected.
	Appearance: Anhedral clasts usually under 100 μm in width.		
Andesine	Distribution: Very sparsely distributed throughout the region.	Not detected in the main sample, although not excluding its presence in other samples.	Not detected
	Appearance: Anhedral clasts usually exceeding 100 μm in width.		
Magnetite	Sparsely distributed throughout all regions.		
	Individual grains range from submicron scale to usually less than 10 μm.		
	Crystal shape superficially similar to apatite, but significantly larger and can be easily distinguished through chemical analysis.		
Ilmenite	Distributed in the mudstone and boundary region albeit very sparsely.		
	Individual grains range from a few microns to less than 20 μm.		

Illite is the main constituent of the hosting mudstone matrix, is the primary clay phase in the boundary region, and is located widely in miniscule pores throughout the bone matrix region. Chemical analysis of illite from every region had minor Mg and Fe content, which may be due to the lower content of tetrahedral and octahedral Al being invariably substituted by Mg and Fe [27,34].

Calcite is an extremely abundant phase from the boundary region and the pores of the rib bone, but its distribution progressively decreases in the mudstone region (S4 Fig and S5B Fig). The richness of calcium carbonate minerals from the fossil site has been well documented [2,3,22]. In the boundary region, very fine grained calcite and clay shows an intermixed distribution without the integration of their constituting elements.

Based on its overall morphology, size, and chemistry, it is suggested that the apatite phase within the bone matrix was diagenetically altered directly from the original bioapatite rather than having an authigenic origin [35].

### Identity of the boundary region and its suggested role in bone preservation

**Fig 9:**
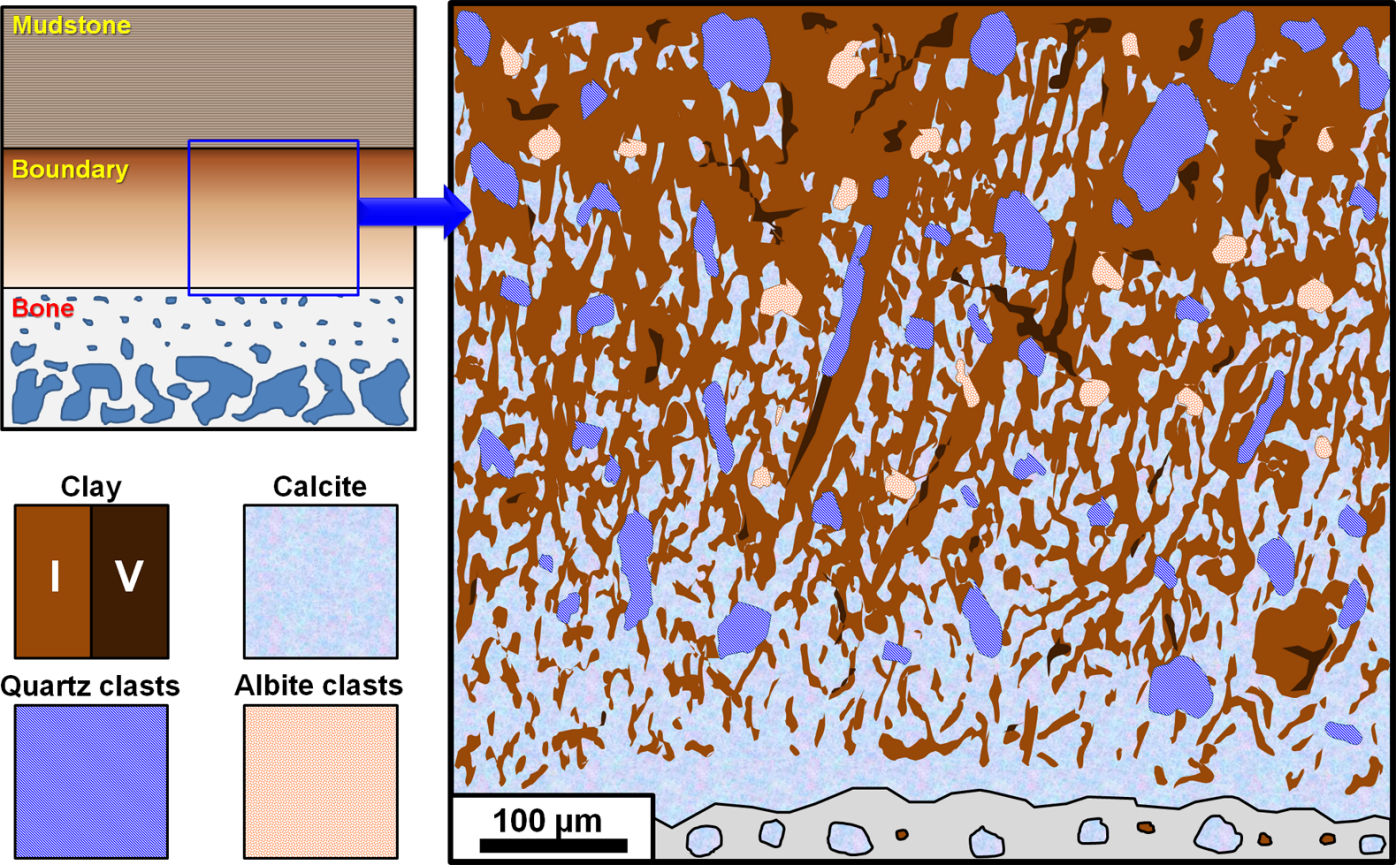
Simplified illustration of the boundary region. The haphazard, reticular orientation of clay and the relatively abundant inclusion of quartz and albite clasts is a notable feature of this region. Clay concentration progressively increases towards the mudstone region, and a thin line of calcite microcrystals covers the bone surface. I = illite, V = vermiculite.

The main phase of the boundary matrix is composed of a mixed distribution of microcrystalline calcite and clay (illite, vermiculite), and may have acted similarly to that of a calcite nodule [36] although we presume that the calcium carbonate content of the region was formed via concretion. Encrustation of calcite on bones is a fairly common feature in Cretaceous fossiliferous localities [23], and is considered to be a significant factor in the preservation of dinosaur bone along with calcareous pedogenesis [2] from the Boseong egg fossil site. In addition, as shown in Fig 9, the distribution pattern of illite and the abundant inclusion of detrital quartz and albite clasts indicate that the region was affected by the rapid accumulation of the hosting mudstone. It is also possible that the increased abundance of calcite content may be related to the bone itself as bone also has high calcium content [37,38]. Although the degree of affinity between calcium carbonate and bone is difficult to quantify, we postulate that the surface of the bone had more direct interaction with calcite, specifically based on the presence of the thin layer of calcite microcrystals on the surface of the bone (Fig 9 and S3 Fig). We also suggest that after full lithification of the region, the fine grained nature of the boundary matrix may have formed a relatively dense and tough shell with low permeability, which may have limited subsequent influx of elements from the mudstone to the bone. Although it is unclear when the boundary region was precisely formed, based on current evidence, we assume that the soluble calcite derived from calcic paleosols [2,23,36] entered the opened spaces around the bone after the decomposition of organic phases while it was still in a buried state and became the foundation of the region. Clay phases within the boundary region were likely derived from the hosting mudstone via precipitation, and also possibly through accumulation when the bone was subject to exposure. The presence of vermiculite interspaced between illite (S9 Fig) is the basis of the latter hypotheses, as vermiculite is typically a byproduct of extensive weathering and is an abundant constituent of soils [25,28]. While the overall hosting mudstone does contain a small amount of vermiculite, based on the chemical mapping data, vermiculite distribution appears to be relatively more concentrated in the boundary region adjacent to the mudstone, which indicates the possibility of an external origin.

### Diagenesis and reaction reflected on illite in the rib bone

The geology of the area which yielded the rib bone fossil has been well investigated and was reported both in local scale [1–5,22] and in larger scale [39,40], although no detailed studies have been reported on clay mineralogy and diagenesis of this area. Regarding to the diagenesis of illite observed in this study, one important factor would be the frequent volcanic activity of the region which is evident from the abundance of tuffaceous rocks in the studied area. The results of our illite study could be summarized as the following: exclusive presence of illite without smectite-illite mixtures, platy morphology of illite crystals, and coexistence of the 1M and 2M illite phases. In this prospect, the origin of the illite phases might be closely related to the dissolution-precipitation process through direct precipitation from the illite-saturated solution, rather than burial formation through the smectite to illite reaction [25,28,31,41–44].

The 2M illite could have either detrital origin or diagenetic origin through the 1M to 2M transformation. Our TEM study strongly suggests the diagenetic origin which requires higher temperature of formation than the 1M illite. It has been reported that the hydrothermal formation of illite usually yields coexistence of the 1M and 2M illite phases [29,41]. The relative abundance of the 1M illite compared to the 2M illite in the studied samples suggested an early stage of the 2M illite formation. High disordering of the 2M illite, especially distinct in the bone region, also supported the diagenetic origin, even though the effect of strain imposed on the illite phases should be considered. Comparatively the 2M illites reported by Lonker and Gerald [29] had much higher lattice ordering than ours, which indicated a more advanced stage of the 2M illite formation as well as a more stable condition of illite formation.

There has been controversy on the mechanisms of the 1M to 2M transformation of illite such as solid-state replacement, simultaneous nucleation and coalescence, or dissolution-precipitation [31]. From previous investigations, the dissolution-precipitation process was suggested to be prevailed in natural hydrothermal systems [31]. More specifically two different views have been suggested on this reaction mechanism; (i) the dissolution of 1M particles and the nucleation and growth of 2M particles [45]; (ii) overgrowth of 2M particles on 1M particles [31]. Composition difference between the 1M and 2M particles were suggested to be in both mechanisms. However, the TEM data that we obtained in this study indicated that there might be another method to achieve the 1M to 2M transformation, that is, solid-state transformation without composition change. The gradual variation of 1M to 2M layer by layer (Fig 5F and S7F Fig) partly supports our view, but may also support the view of the overgrowth of 2M on 1M. Instead, the lateral variation of 1M to 2M within the same layer (Fig 6) strongly supported our view of solid-state transformation without composition change. We definitely need further study to verify that this mechanism prevails more in our system as well as other systems.

### Preservation of the rib bone matrix

The well-articulated nature of the holotype specimen KDRC-BB2 indicates a rapid burial process [2, 4,46]. Prolonged surface exposure would have left the specimen vulnerable to scavenging and bone separation due to bloating and decomposition of the organic tissues [46]. Although the surface of the skeletal remains showed partial erosion likely due to exposure and weathering for certain periods of time post-fossilization, we found no discernible teeth marks or features indicating bioturbation [4]. Therefore, we suggest that rapid burial in a terrestrial environment was the initial process experienced by KDRC-BB2. Although the fossil site was close to channels and was subject to periodic flooding, the development of vertic and calcic palaeosols indicates that flooding did not happen frequently, and was probably a seasonal event [2].

While in a buried state, the organic matter on both the surface and within the bone started to gradually decay, leaving open spaces. It is also possible that microbial activity may have taken effect on bones by facilitating bioerosion and compositional alterations [47]. After organic decomposition, the open spaces provided a means of influx of solutions rich in elements derived from the depositional environment. It is likely that clay elements, specifically illite, had the most interaction with the bone matrix itself. We suggest that illite entered the pores of the bone through dissolution and precipitation. Illite was especially rich in regions with lower bone matrix density and higher porosity.

While illite was occupying almost every pore smaller than 20~30 μm in width, those exceeding 40 μm in width were predominantly filled with calcite microcrystals. This feature strongly indicates that illite had passed through the pores, which allowed calcite to occupy the larger open spaces instead. It is also possible that some of the slightly larger spaces may have been initially filled with illite, albeit in a less dense and a more random distribution, making it easier for other phases to push it out. Calcic paleosols within the hosting mudstone may likely have been a significant and direct source of calcite to the bone. It has been suggested that calcareous pedogenesis may have assisted in the preservation of dinosaur egg fossils previously discovered at the site [2]. The occurrence of clusters of calcite microcrystals in the pore spaces of the hosting mudstone and in the pores within the fossil bone indicates that calcite crystals were formed in similar, possibly in identical conditions. Calcite microcrystals were also found in various cracks throughout the bone matrix. The lack of calcite in smaller pores containing illite strongly suggests that calcite did not penetrate nor occupy such constrained spaces. The formation of calcite microcrystal clusters is due to the subsequent dehydration and extensive, gradual pressure [36] applied by the depositional environment.

TEM analysis from the select FIB samples shows that the fossilized apatite retained varying degrees of preferred orientation along the long axis of the rib bone, which reinforces the hypothesis that the fossil apatite diagenetically originated from bioapatite as the hierarchical structure of biological bone [47–50] was reflected. Based on Fig 7, cross-FIB-milled samples from bone matrix areas rich in illite show higher degrees of preferred apatite orientation, suggesting that illite may have taken a direct role in preserving the structural integrity of the bone. Samples lacking illite show a notably lower degree of or no preferred orientation. Therefore, we can infer that the distribution of illite helped increase the overall density of bone, thus increasing the possibility of localized nanostructural preservation. Such features were also observed from a highly porous bone matrix region from the left femur of *Koreanosaurus*, indicating the same role in local nanostructure preservation [18].

The weathered nature of the surface of the bone, the presence of vermiculite in the boundary region, and the proposed cycling of wet and dry seasons of the site [2] constitute strong evidences that the skeletal elements of KDRC-BB2 were subject to exposure. The lack of vermiculite within the bone matrix, and the articulated state of KDRC-BB2 implies that the initial fossilization process occurred during burial and that the bone was probably not subject to periodic exposure prior to full fossilization. As KDRC-BB2 represents mostly the “torso” region of the individual, other skeletal elements may have been lost during exposure or due to local geological factors such as volcanic activities [1–4,39,40].

Besides rapid burial, we suggest that the increase in the bone matrix density owing to the occupation of illite crystals in its miniscule pores; crystallized calcite occupying its larger pores throughout the bone; and the diagenetic formation of fluorapatite [50] all played a role in the overall preservation of the rib bone, and the holotype specimen in general. In addition, the formation of the boundary region may have protected the fossil bone from interaction of other elements and further erosion when exposed.

### Osteohistological preservation of *Koreanosaurus*

**Fig 10:**
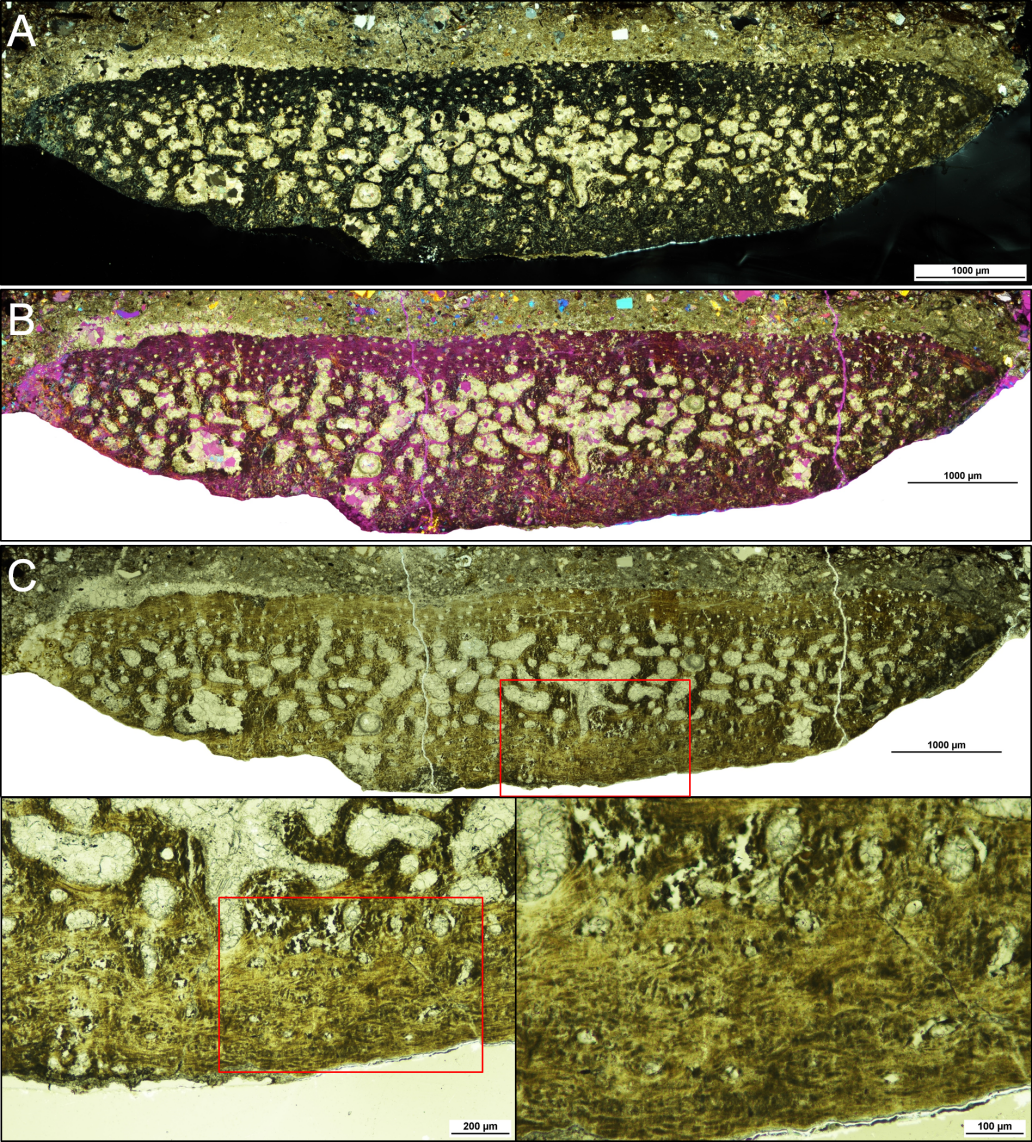
Composite optical micrographs of the main thin section under different light settings. (A) Cross-polarized light. (B) Polarized light with the lambda wave plate (530 nm) inserted. (C) Normal transmitted light. Although poorly preserved, osteohistological features are best observed under normal transmitted light as shown in the magnified images in (C).

Although the skeletal elements of *Koreanosaurus* were sufficiently preserved for evaluating diagnostic anatomical features [4], the osteohistological features of the rib bone were considerably hindered (Figs 1 and 10 and S10 Fig). We have initially suspected that this issue might have stemmed from optical thin sections being too thick, but after preparing several thin sections in varying degrees of thickness (S10 Fig), it has become quite evident that even at an optimum sample thickness (S10C Fig), the overall microstructural preservation of the rib bone was observed to be poor. This feature is consistent with the dorsal rib portion from our previous study [17], and also with the left femur [18].

Based on our structural data from the rib bone and femoral bones so far, it is very likely that extensive pressure was the key factor of hindering osteohistological details. At macroscale, the warping of individual rib bones serves as key evidence of applied pressure, and various cracks throughout the rib bone in both macro- and microscale provides further evidence of high pressure. Most of the skeletal elements from KDRC-BB3 (the paratype specimen which includes the left femur) also showed macroscale features indicating that the skeletal elements were subject to high pressure [4,18].

As shown in Fig 10C, although the arrangement of neurovascular channels is intact, we cannot specifically identify the main cortical bone tissue type as the distribution pattern of osteocyte lacunae is unclear. The osteohistological features of the left femur had a similar level of hindrance, and bone matrix regions disrupted by calcite intrusions had worse preservation levels [18] compared to the rib bone. The peculiar preservation feature of both the rib bone and left femur is that both samples had certain bone matrix regions with high degrees of nanostructural preservation, despite poor microstructural preservations [18]. As KDRC-BB2 and KDRC-BB3 were discovered at the same locality (Site 5) and at an almost identical state (directly embedded in thick mudstone), we can presume that both specimens underwent analogous fossilization processes, which resulted with overall similarities in preservation levels from macro- to nanoscale. On the other hand, the right femur from the referred paratype specimen KDRC-BB1 displayed opposite levels of preservation compared to the rib bone and the left femur. While the right femur had relatively well-preserved osteohistological features, the nanostructural preservation levels were generally poor which may be attributed to both geological and biological factors [18]. The femur was discovered inside a calcite nodule at Site 3 and was likely not subject to extensive pressure and expedited diagenesis during initial fossilization [4,18], and the wide distribution of woven bone tissue may have also affected apatite crystal organization although further study is required for verification [18]. Therefore, based on the rib bone and left femur, we suggest that the increased density of the affected bone matrices via extensive pressure, rapid rate of diagenetic alteration of bioapatite crystals, and the inclusion and diagenesis of illite may have preserved the bone nanostructure while osteohistological features became hindered.

## Conclusions

Using correlative microscopy, we identified the key constituents from a dinosaur fossil rib bone and its hosting mudstone. Our results revealed that illite and calcite were the key overlapping phases in the hosting mudstone, the boundary, and the bone matrix region. Illite was the main constituent of the hosting mudstone, and also frequently occurred in the boundary region and in miniscule pores throughout the rib bone matrix. Calcite primarily existed in clusters of microcrystals with various orientations in the larger pore spaces. The very fine-grained and densely packed nature of both calcite and clay in the boundary region matrix may have inhibited the permeability between the bone and the hosting mudstone, and thus possibly aided the preservation of bone. The apatite phase of the rib bone was fluorapatite, and we suggest that it has diagenetically originated from bioapatite. Although clay phases occupying vascular channels in fossil bone have been previously documented, we have revealed unprecedented details of the structural properties of illite occupying minicule pores of the rib bone matrix through EM. The platy morphology, arrangement, and crystallography of illite within the bone matrix indicate that it was formed possibly by dissolution-precipitation process. TEM study of illite within the bone matrix indicated the solid-state transformation of 1M to 2M without composition change, which was more evident from the HRTEM image showing the lateral variation of 1M to 2M within the same layer. The high level of lattice disordering of 2M suggested an early stage of 1M to 2M transformation. Based on our data, we suggest that the increased crystallinity and dimension of diagenetically altered apatite and illite crystals may have directly taken a role in preserving the bone over geologic time scales. This may have been achieved by the increased density of the bone matrix while decreasing the number of open spaces, thus making the rib bone capable of withstanding high pressure and preserving some of its nanostructures despite the osteohistological features being poorly-preserved. It is quite clear that rapid burial was a key process in bone preservation based on large-scale geological features of the local site and the articulated state of KDRC-BB2. However, albeit from a small bone portion, deeper investigation of factors involved in bone preservation such as the interaction of key elements, formation of the boundary region, and the diagenetic alteration of apatite and illite within the bone matrix cannot be thoroughly evaluated without correlative microscopy.

## Supporting information

S1 Fig The holotype specimen of *Koreanosaurus boseongensis*. (A) Left dorsolateral view of the specimen. The arrow indicates the sampled rib bone. (B) Distal portion of the seventh left dorsal rib bone. The arrow indicates the region where the main optical thin sections were prepared. The region below the yellow line was sliced off and was powdered for comparative XRD analysis.
(TIF)

S2 Fig Schematics of the ultrasonic drilling and spraying devices. (A) Ultrasonic drill, with drill tip specifications. (B) Ultrasonic sprayer for dispersing powdered samples, which is capable of loading up to 4 samples on separate areas of the TEM grid via a masking tool. (C) Illustration of the overall process of drilling, powder collecting and dispersing on the TEM grid.
(TIF)

S3 Fig Optical micrographs of calcite clusters (red arrows) from the hosting mudstone, boundary and rib bone regions. The inset on the upper right is a simplified illustration of the calcite microcrystals in various orientations. Note that distinct clusters of calcite microcrystals occur on the boundary region where clay content is lacking.
(TIF)

S4 Fig EPMA mapping from adjacent regions–mudstone/boundary (EPMA 1), boundary (EPMA 2), and boundary/rib bone (EPMA 3). The distribution of calcite is sharply increased in the boundary region (EPMA 1), and the clay phases does not overlap with calcite indicating that these phases were not intermixed (EPMA 2). The most adjacent area between the boundary and bone matrix shows a high concentration of calcite distribution. P occurs nearly exclusively in the bone matrix, and Al, Si, K are distributed within the bone matrix, but are highly concentrated near the small cracks and openings. Larger openings are mostly filled with calcite (EPMA 3). Mg spiking regions overlapping with Al and Si, while notably lacking in K indicates the distribution of vermiculite. Due to the presence of a bone fragment, magnetite clasts and small quartz clasts, EPMA 3 was mapped intentionally out of alignment from EPMA 1 & 2. BEI = backscattered electron image.
(TIF)

S5 Fig SEM analysis. (A) Illite occupying a small vascular channel within the rib bone region. (B) EDS mapping of the main optical thin section. The concentrated distribution of Mg (blue arrow) is an intriguing feature from the area where the boundary and mudstone meets. Note that Na, Mg and Fe from the rib bone region are noise signals rather than representing the distribution of these elements.
(TIF)

S6 Fig Optical thin section for FIB milling. This section directly faced the main thin section. FIB samples were prepared from the designated locations and visible clasts were avoided. EDS point scans were performed before marking specific areas for FIB milling. C = cross, P = plane, 1–3: Boundary region (1—adjacent to mudstone, 2 –middle region, 3—adjacent to rib bone). 4: Mudstone matrix. 5: Rib bone matrix.
(TIF)

S7 Fig Additional HRTEM images and SAED patterns from the designated areas (yellow circled) in TEM micrographs of clay : From mudstone ((A), (B)), boundary ((C), (D)), and bone ((E), (F)) regions. The parallel boundaries between 1M and 2M illites are arrowed in (B) and (F). The parallel boundary between illite and vermiculite is arrowed in (A). Note the gradual boundaries between 1M and 2M, and the highly disordered {00l} 2M lattices in (B) and (F).
(TIF)

S8 Fig TEM micrographs and SAED patterns of apatite crystals from the designated areas (yellow circles) of FIB 3–5 and FIB 7. All diffraction data obtained from the plane samples lacked preferred orientation of apatite crystals. C = calcite, I = illite.
(TIF)

S9 Fig Vermiculite wedged within illite distrbution at the boundary region. In EDS maps, strong K signals represents illite, and strong Mg signals represents vermiculite. (A) OM and SEM micrographs and the corresponding SEM-EDS mapping data (x2K) indicating the sampled area. (B) TEM-EDS mapping data of the FIB-milled sample (cross section) from the area designated by the yellow arrow in (A). I = illite, V = vermiculite.
(TIF)

S10 Fig Investigating the optimum thickness for observing osteohistological features from optical thin sections. (A) An optical thin section sample with different thickness levels. (B) An optical thin section sample with an overall thickness around 70 μm. (C) Identical sample to (B) with an overall thickness around 40 μm. As shown in the magnified image of the inset, poorly preserved osteohistological features can be clearly observed.
(TIF)

S1 Table Sedimentary sequence of the Boseong fossil site. (DOCX)

S2 Table The interplanar spacings and chemistry of clay phases from FIB-milled samples. (DOCX)
