# Correction: Correlative microscopy of the constituents of a dinosaur rib fossil and hosting mudstone: Implications on diagenesis and fossil preservation

**DOI:** 10.1371/journal.pone.0195421

**Published:** 2018-03-29

**Authors:** Jung-Kyun Kim, Yong-Eun Kwon, Sang-Gil Lee, Chang-Yeon Kim, Jin-Gyu Kim, Min Huh, Eunji Lee, Youn-Joong Kim

There are errors in the Funding section. The correct funding information is as follows: This study was supported by the National Research Foundation of Korea Grant funded by the Korean Government (MSIP) (2017, R&D Equipment Engineer Education Program, 2014R1A6A9064166) and by the Korea Basic Science Institute (institutional program: D37612).

There is an error in the fourth sentence of the "XRD" section within the Methods. The correct sentence is: For the finalized data, we analyzed identical samples with an X’Pert-PRO XRD (PANalytical) with 2θ ranges of 5° to 90°, a step of 0.013°, and a step duration of 30 s.

There is an incorrect reference in the fifth sentence of the fourth paragraph within the "TEM" section of the Results. Reference 1 should not be cited.

In Table 2, the "Distribution" in the Rib Bone column for Feldspars Albite incorrectly refers to EM when it should refer to TEM. Please see the corrected Table 2 here.

**Table 2:** Identified phases and their distributions in each region of the samples studied.

Region Phase	Mudstone	Boundary	Rib Bone
Apatite (Fluorapatite)	Distribution: Only tiny specs of phosphorous were detected, thus apatite is virtually absent in the hosting mudstone.	Distribution: Detected only from a very small number of bone fragments in the region closely adjacent to the bone surface.	Distribution: Main phase of the bone matrix region.
			Appearance: Composed of tightly packed euhedral fluorapatite crystals ranging from 80~200 nm. Varying degrees of preferred orientation along the long axis of the bone
Calcite	Distribution: Widely distributed throughout the entire region as clusters of calcite microcrystals occupying the pore spaces.	Distribution: Widely distributed throughout the entire region and is the main constituent of the matrix.	Distribution: Widely distributed as clusters of calcite microcrystals filling pores and cracks in the bone.
	Appearance: Individual microcrystals usually around a few microns to 30 μm of varying orientations tightly packed in clusters mostly under 200 μm. Only a few clusters exceed 300 μm in width.	Appearance: Individual crystals of submicron to a few microns scale prominent throughout regions with rich clay content. A thin cluster of calcite microcrystals nearly 2 mm of length adjacent to the bone region is composed of microcrystals usually around a few microns to 30 μm.	Appearance: Individual microcrystals usually around a few microns to 30 μm tightly packed in clusters correlating to the size of the pores. Pores typically exceeding 40 μm are filled with calcite microcrystals.
Clays Illite	Distribution: Main constituent of the mudstone matrix.	Distribution: Widely distributed and secondary constituent of the matrix.	Distribution: Widely distributed in smaller pores and the matrix.
	Appearance: Exists as thin plates of varying orientations. Illite crystals occupy even extremely constrained spaces.	Appearance: Exists as thin plates of varying orientations. Usually wedged between larger calcite crystals.	Appearance: Exists as thin plates of varying orientations.
Vermiculite	Distribution: Minor clay phase throughout the matrix. Detected and identified through XRD analysis.	Distribution: Secondary clay phase, concentrated near the mudstone.	Not detected.
		Appearance: Exist as thin plates of varying orientations.	
Kaolinite	Distribution: Minor clay phase throughout the matrix. Detected and identified through XRD analysis.	Not detected.	Not detected.
Quartz	Distribution: Widely distributed throughout the entire region as clastic grains, and is the most abundant visible grain.	Distribution: Most widely distributed among visible detrital grains.	Distribution: Sparsely distributed throughout the matrix region (XRD), directly observed by EM.
	Appearance: Individual grains range from a few μm to over 300 μm in width and are in varying orientations. All grains are anhedral and grains exceeding 200 μm are sparse.	Appearance: Individual grains of varying orientations range from a few microns to over 200 μm in width although mostly under 50 μm. All grains are anhedral.	Appearance: Individual crystals with virtually round morphology in submicron scale. Usually around a few hundred nanometers.
Feldspars Albite	Distribution: Widely distributed throughout the region	Distribution: Common throughout the region, secondary clast phase.	Distribution: Sparse throughout the region (XRD), not directly observed by TEM.
	Appearance: Anhedral grains usually around and under 50 μm in width.	Appearance: Anhedral grains usually around and under 30 μm in width.	Appearance: Grains around or under 10 μm.
Sanidine	Distribution: Moderately distributed throughout the region, secondary feldspar phase.	Not detected in the main sample, was not discernible through chemical mapping due to overlapping chemical elements with illite.	Not detected.
	Appearance: Anhedral clasts usually under 100 μm in width.		
Andesine	Distribution: Very sparsely distributed throughout the region.	Not detected in the main sample, although not excluding its presence in other samples.	Not detected
	Appearance: Anhedral clasts usually exceeding 100 μm in width.		
Magnetite	Sparsely distributed throughout all regions.		
	Individual grains range from submicron scale to usually less than 10 μm.		
	Crystal shape superficially similar to apatite, but significantly larger and can be easily distinguished through chemical analysis.		
Ilmenite	Distributed in the mudstone and boundary region albeit very sparsely.		
	Individual grains range from a few microns to less than 20 μm.		

The second sentence of the fourth paragraph in the "Preservation of the rib bone matrix" section of the Discussion should have cited Fig 8 instead of Fig 7. The correct sentence should read: Based on Fig 8, cross-FIB-milled samples from bone matrix areas rich in illite show higher degrees of preferred apatite orientation, suggesting that illite may have taken a direct role in preserving the structural integrity of the bone.
