# Supplementary figures and images for: Correlative microscopy of the constituents of a dinosaur rib fossil and hosting mudstone: Implications on diagenesis and fossil preservation
Source: PLoS One. 2017 Oct 19;12(10):e0186600. doi: 10.1371/journal.pone.0186600 (PMC5648225; doi:10.1371/journal.pone.0186600)

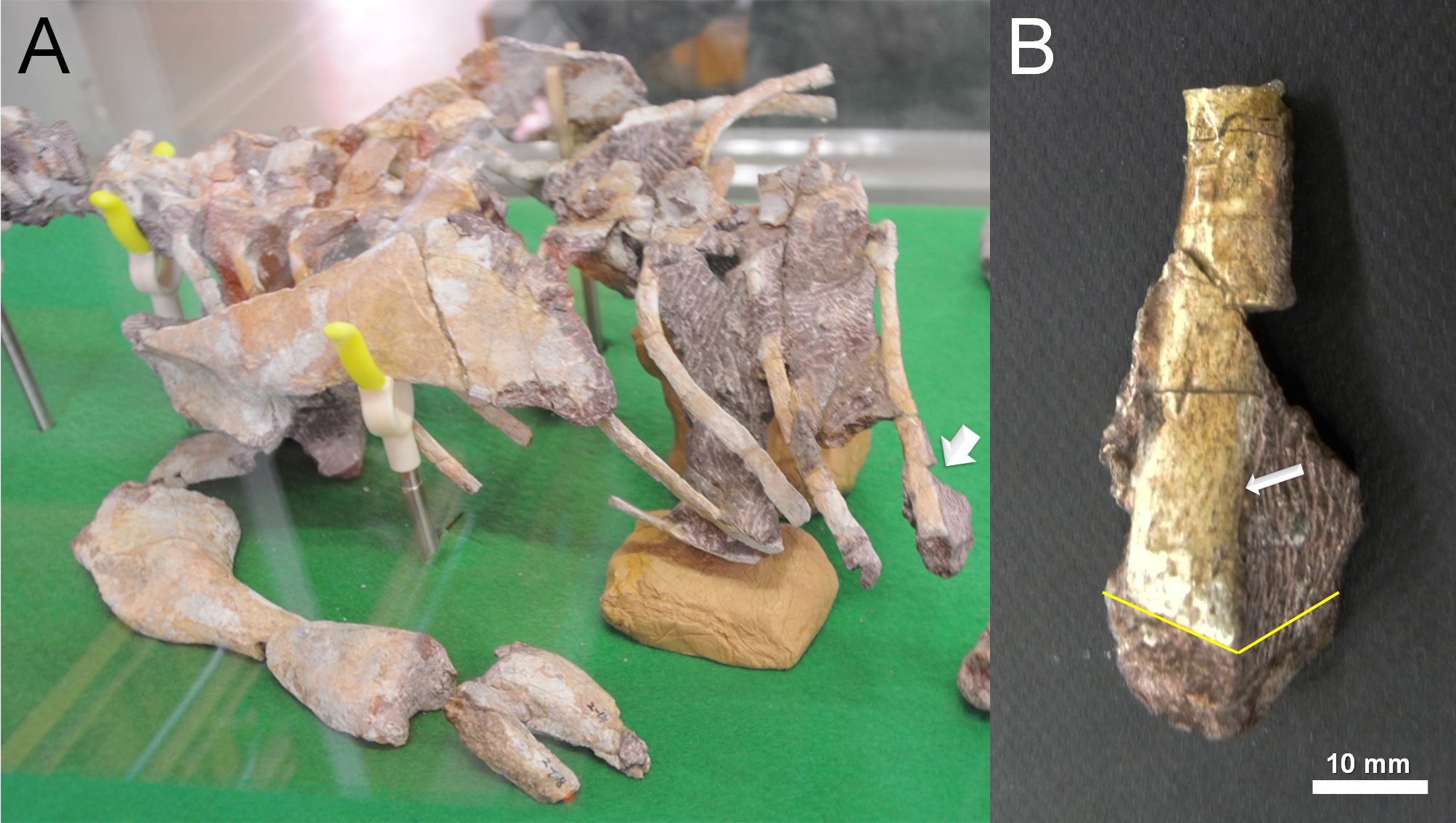

Supplement: S1 Fig — (A) Left dorsolateral view of the specimen. The arrow indicates the sampled rib bone. (B) Distal portion of the seventh left dorsal rib bone. The arrow indicates the region where the main optical thin sections were prepared. The region below the yellow line was sliced off and was powdered for comparative XRD analysis. (TIF) [file pone.0186600.s001.tif]

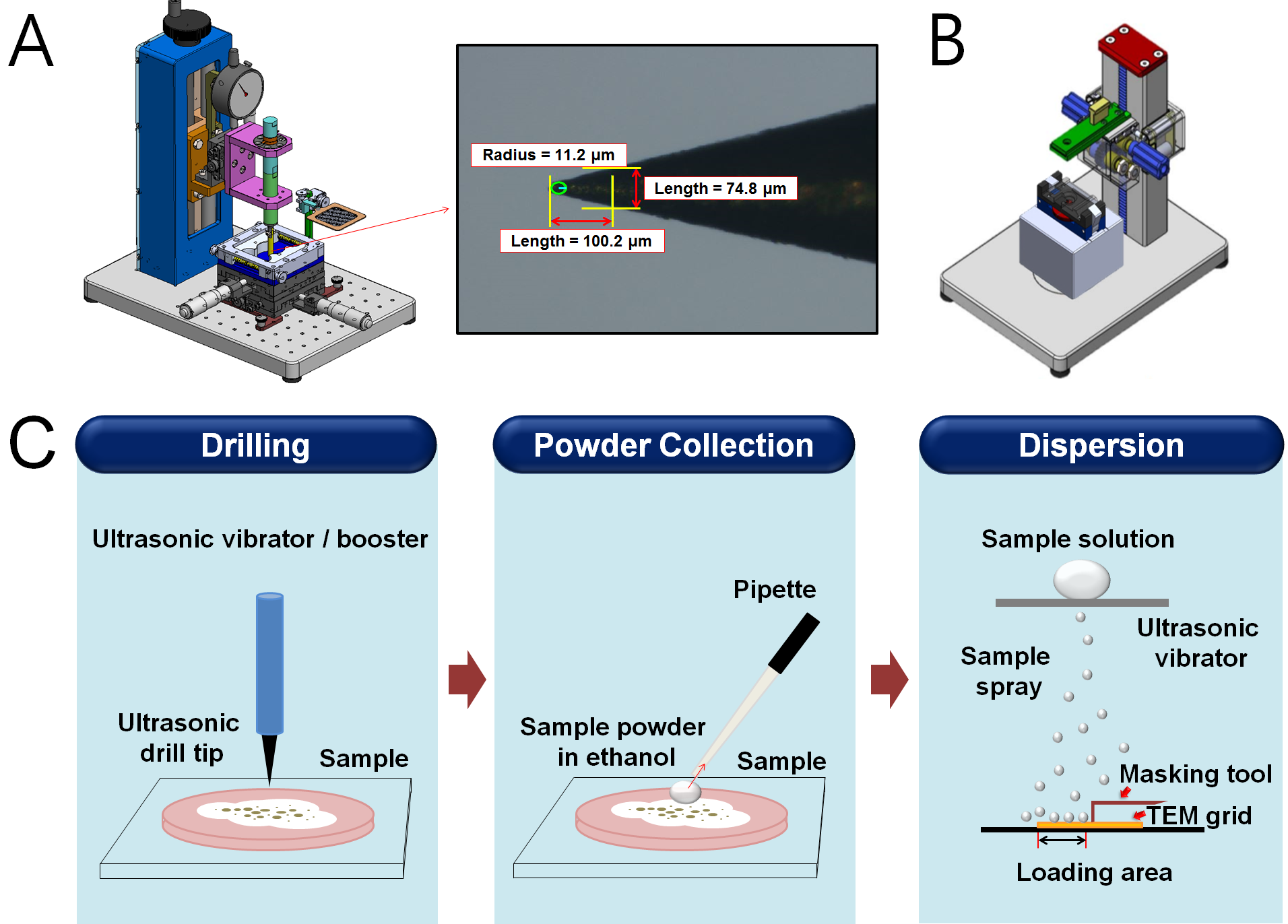

Supplement: S2 Fig — (A) Ultrasonic drill, with drill tip specifications. (B) Ultrasonic sprayer for dispersing powdered samples, which is capable of loading up to 4 samples on separate areas of the TEM grid via a masking tool. (C) Illustration of the overall process of drilling, powder collecting and dispersing on the TEM grid. (TIF) [file pone.0186600.s002.tif]

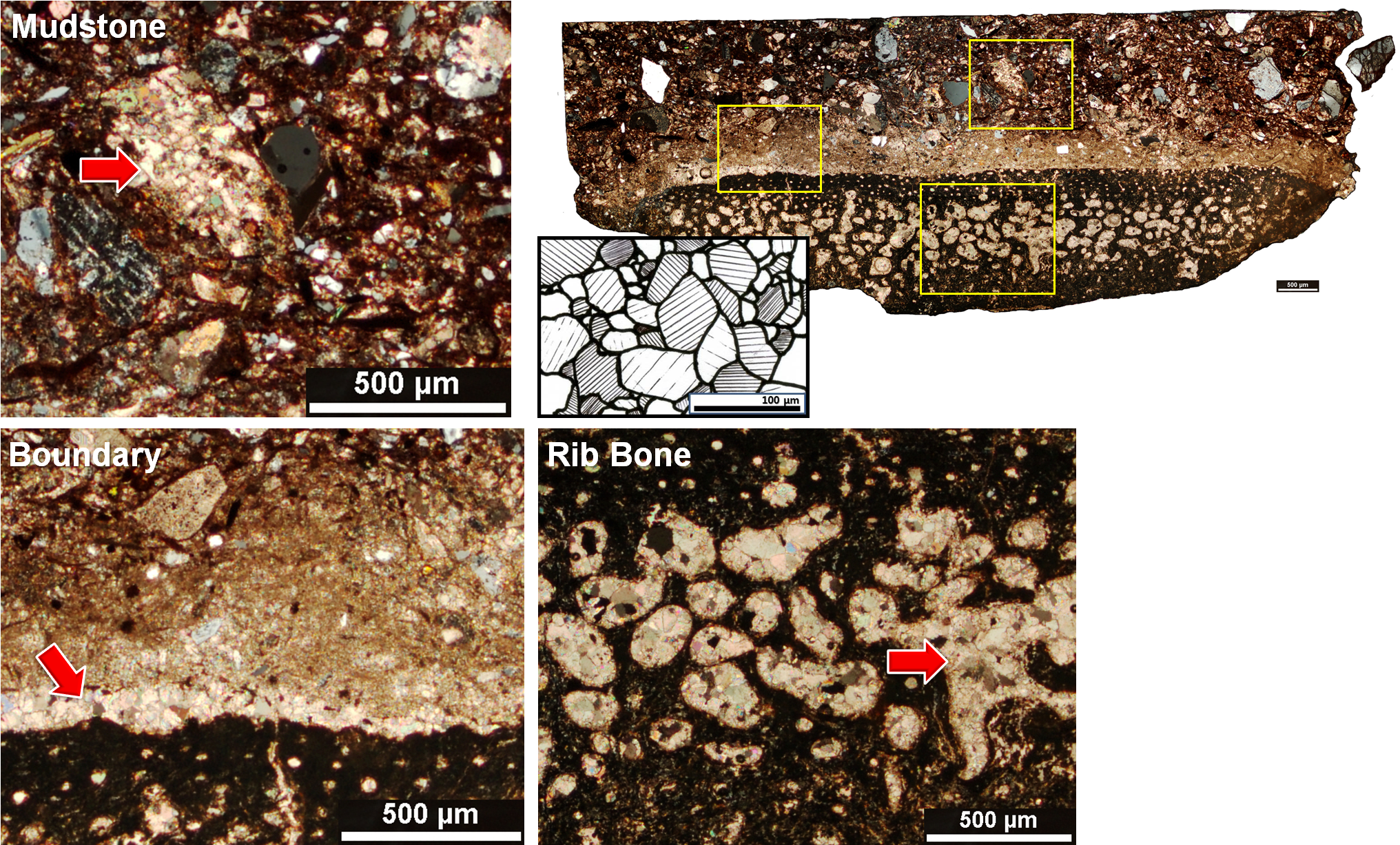

Supplement: S3 Fig — The inset on the upper right is a simplified illustration of the calcite microcrystals in various orientations. Note that distinct clusters of calcite microcrystals occur on the boundary region where clay content is lacking. (TIF) [file pone.0186600.s003.tif]

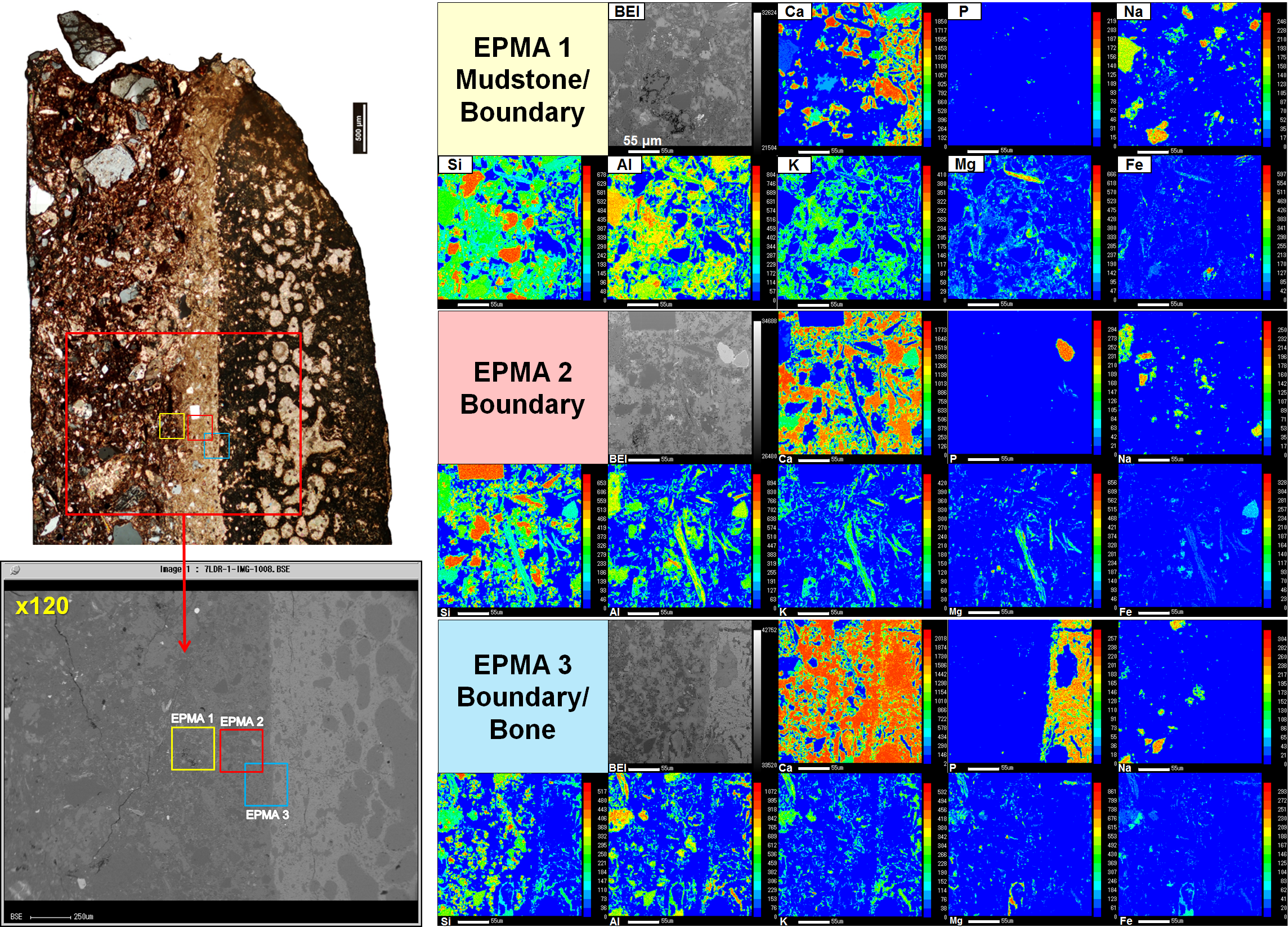

Supplement: S4 Fig — The distribution of calcite is sharply increased in the boundary region (EPMA 1), and the clay phases does not overlap with calcite indicating that these phases were not intermixed (EPMA 2). The most adjacent area between the boundary and bone matrix shows a high concentration of calcite distribution. P occurs nearly exclusively in the bone matrix, and Al, Si, K are distributed within the bone matrix, but are highly concentrated near the small cracks and openings. Larger openings are mostly filled with calcite (EPMA 3). Mg spiking regions overlapping with Al and Si, while notably lacking in K indicates the distribution of vermiculite. Due to the presence of a bone fragment, magnetite clasts and small quartz clasts, EPMA 3 was mapped intentionally out of alignment from EPMA 1 & 2. BEI = backscattered electron image. (TIF) [file pone.0186600.s004.tif]

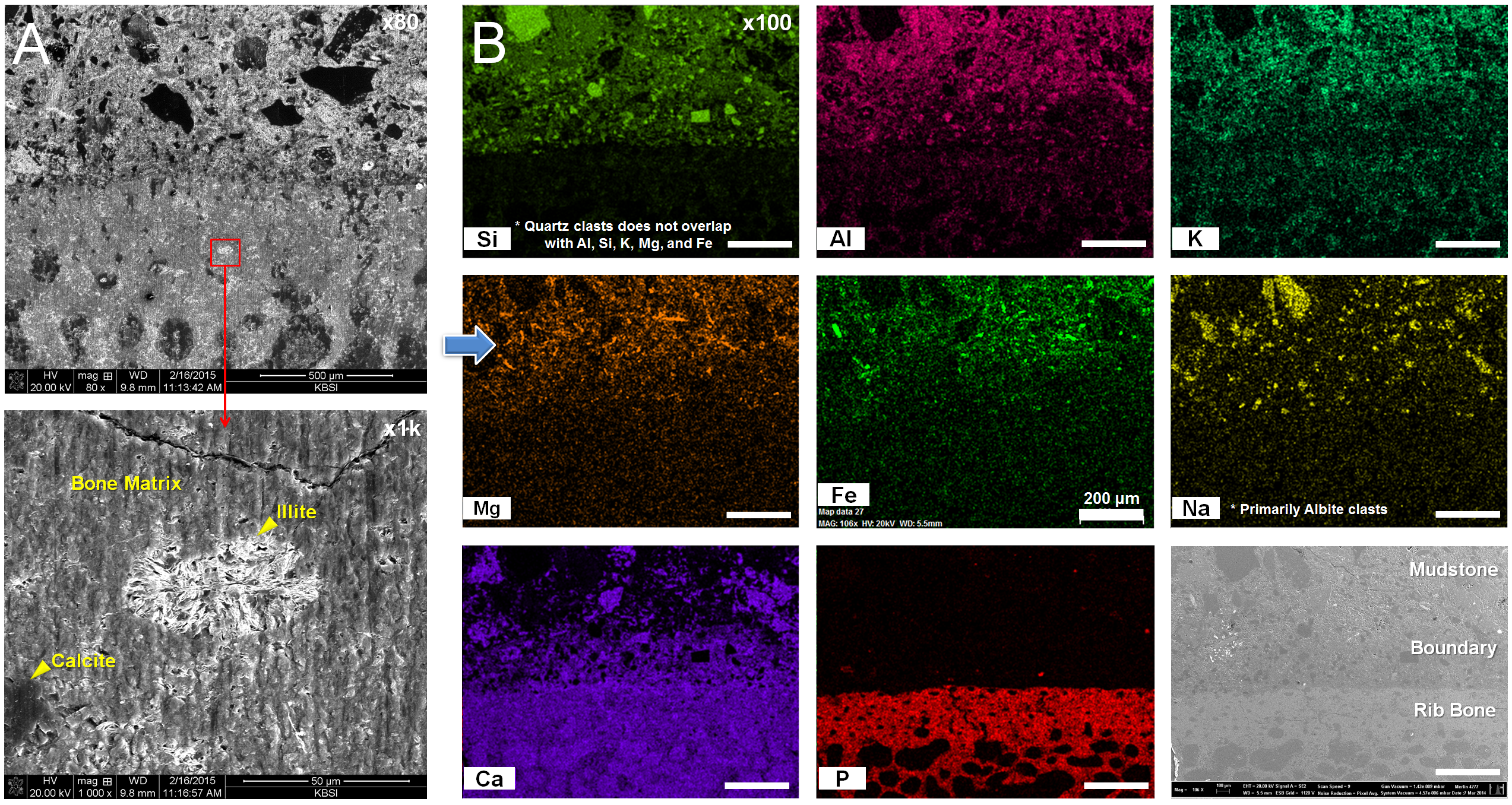

Supplement: S5 Fig — (A) Illite occupying a small vascular channel within the rib bone region. (B) EDS mapping of the main optical thin section. The concentrated distribution of Mg (blue arrow) is an intriguing feature from the area where the boundary and mudstone meets. Note that Na, Mg and Fe from the rib bone region are noise signals rather than representing the distribution of these elements. (TIF) [file pone.0186600.s005.tif]

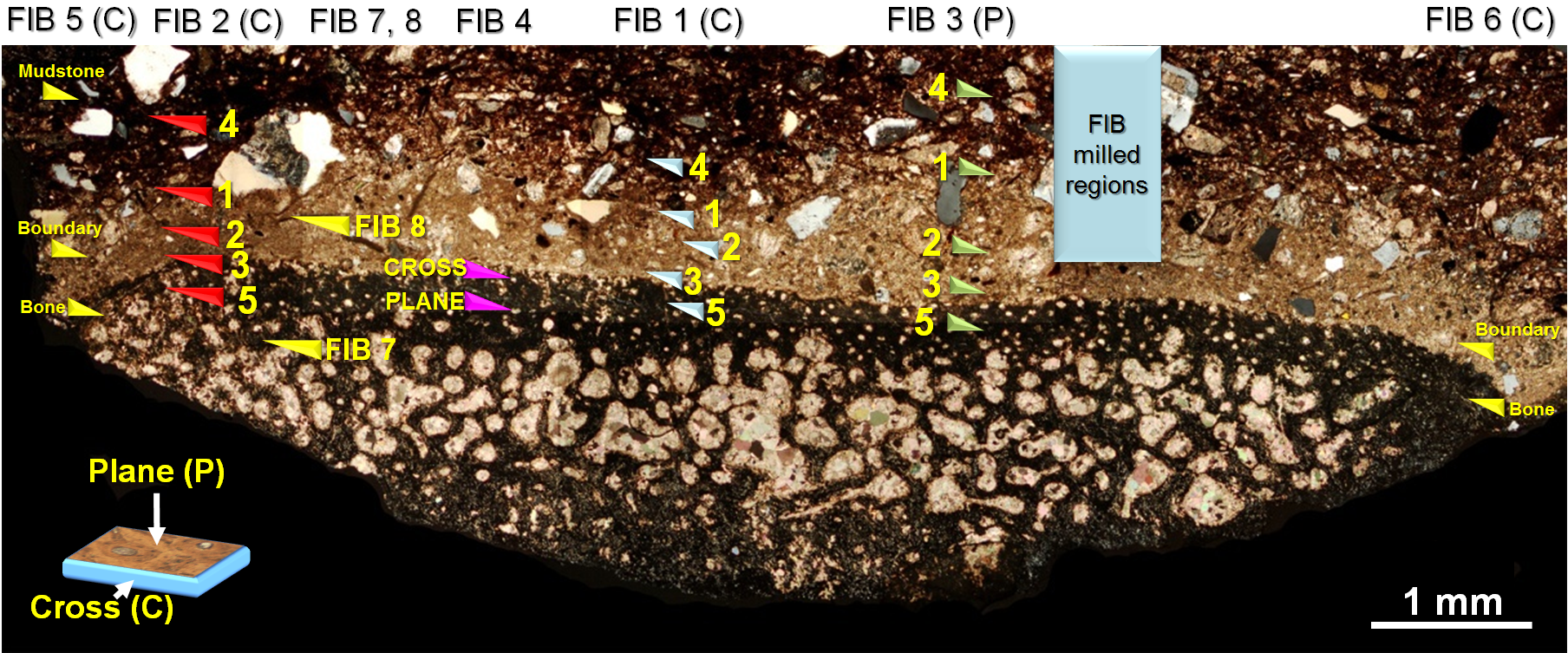

Supplement: S6 Fig — This section directly faced the main thin section. FIB samples were prepared from the designated locations and visible clasts were avoided. EDS point scans were performed before marking specific areas for FIB milling. C = cross, P = plane, 1–3: Boundary region (1—adjacent to mudstone, 2 –middle region, 3—adjacent to rib bone). 4: Mudstone matrix. 5: Rib bone matrix. (TIF) [file pone.0186600.s006.tif]

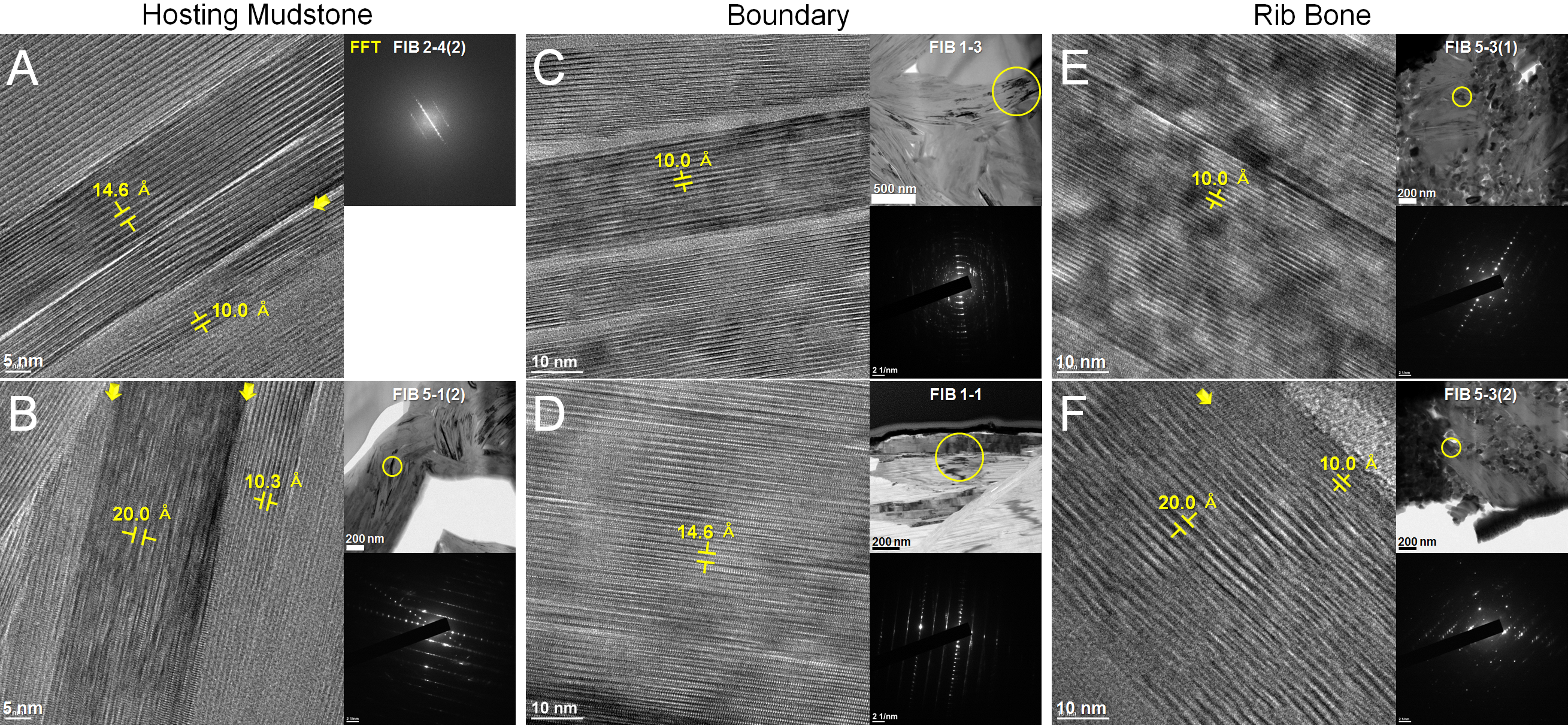

Supplement: S7 Fig — : From mudstone ((A), (B)), boundary ((C), (D)), and bone ((E), (F)) regions. The parallel boundaries between 1M and 2M illites are arrowed in (B) and (F). The parallel boundary between illite and vermiculite is arrowed in (A). Note the gradual boundaries between 1M and 2M, and the highly disordered {00l} 2M lattices in (B) and (F). (TIF) [file pone.0186600.s007.tif]

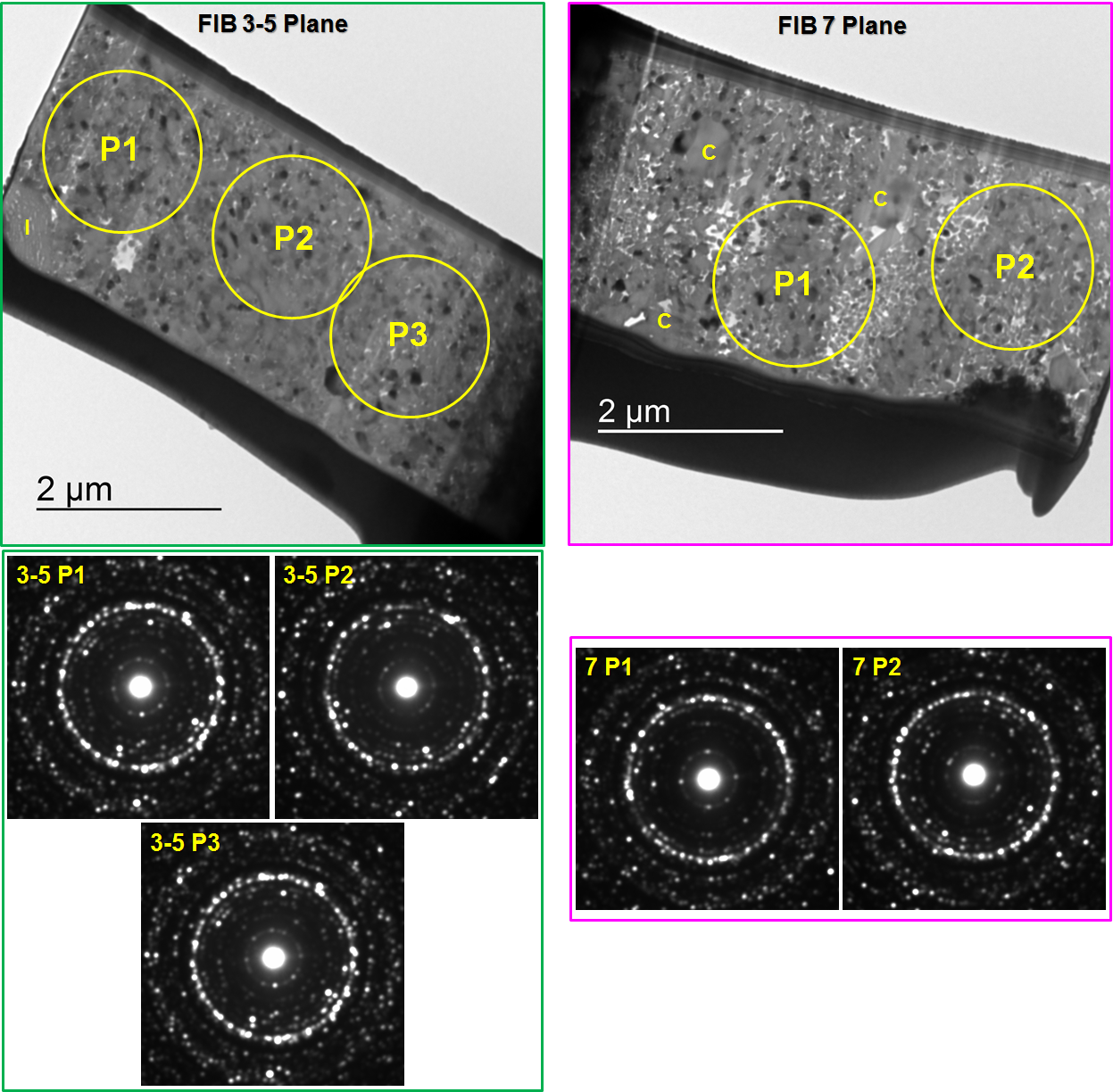

Supplement: S8 Fig — All diffraction data obtained from the plane samples lacked preferred orientation of apatite crystals. C = calcite, I = illite. (TIF) [file pone.0186600.s008.tif]

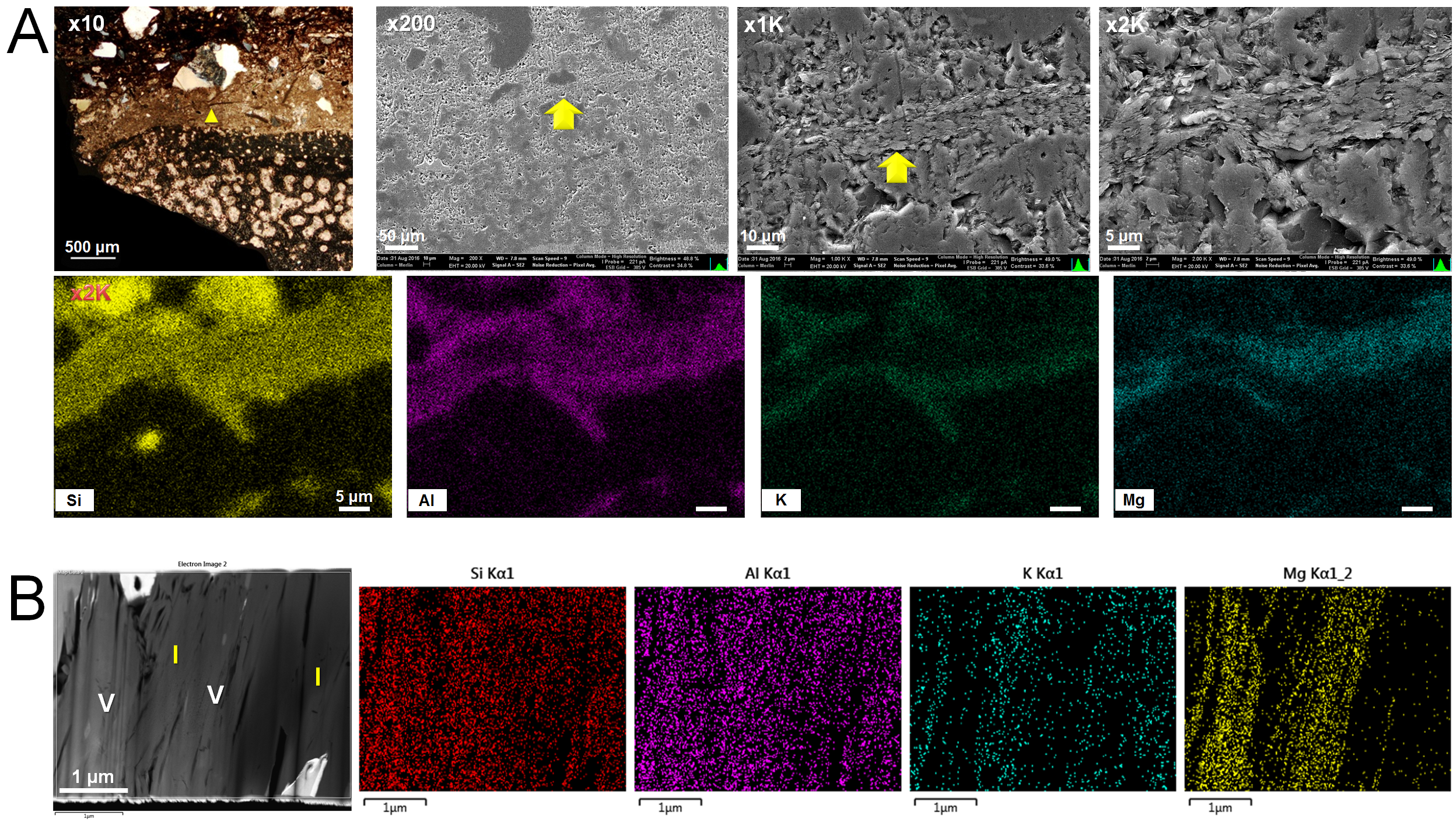

Supplement: S9 Fig — In EDS maps, strong K signals represents illite, and strong Mg signals represents vermiculite. (A) OM and SEM micrographs and the corresponding SEM-EDS mapping data (x2K) indicating the sampled area. (B) TEM-EDS mapping data of the FIB-milled sample (cross section) from the area designated by the yellow arrow in (A). I = illite, V = vermiculite. (TIF) [file pone.0186600.s009.tif]

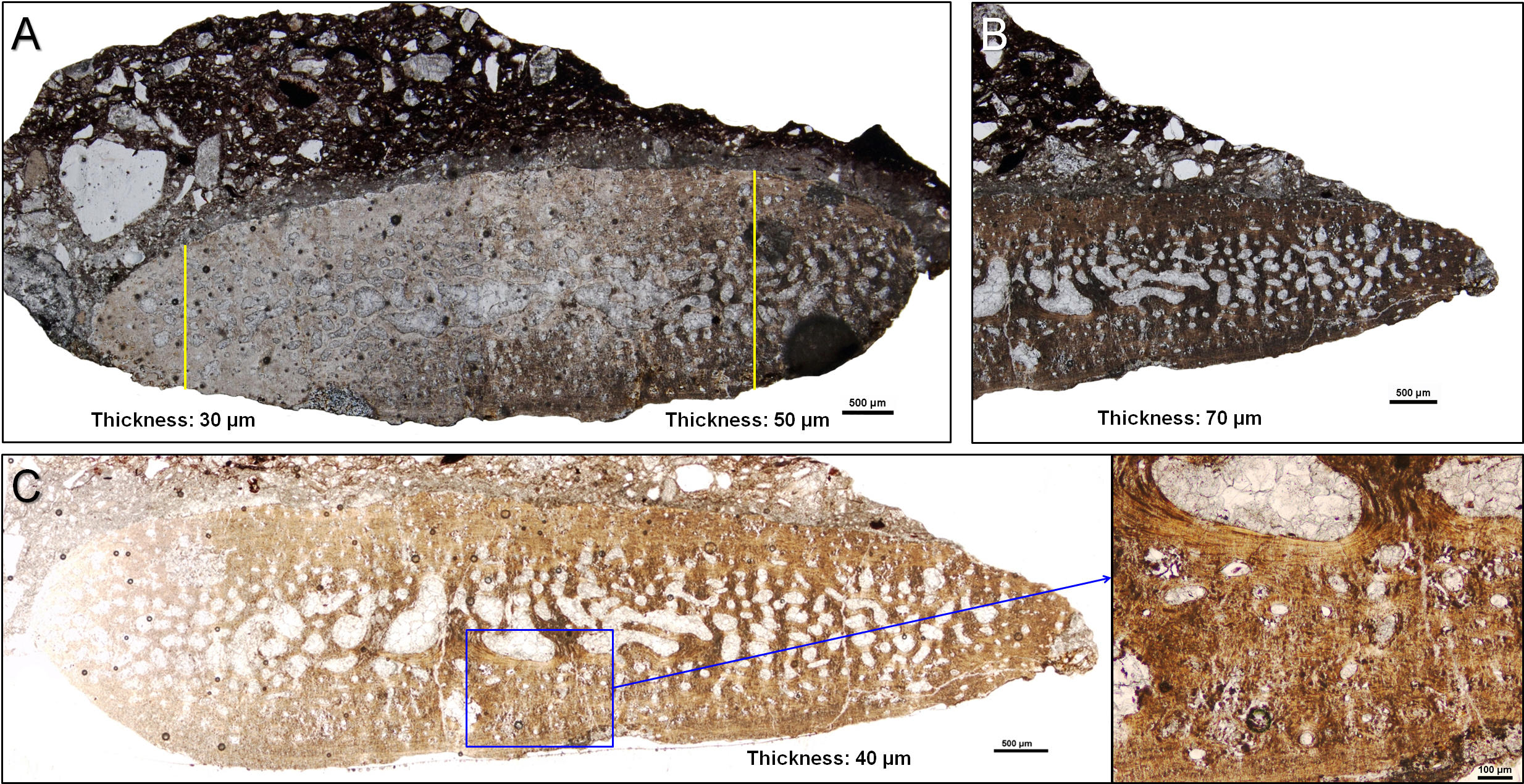

Supplement: S10 Fig — (A) An optical thin section sample with different thickness levels. (B) An optical thin section sample with an overall thickness around 70 μm. (C) Identical sample to (B) with an overall thickness around 40 μm. As shown in the magnified image of the inset, poorly preserved osteohistological features can be clearly observed. (TIF) [file pone.0186600.s010.tif]
