# Supplementary material for: Correlative microscopy of the constituents of a dinosaur rib fossil and hosting mudstone: Implications on diagenesis and fossil preservation
Source: PLoS One. 2017 Oct 19;12(10):e0186600. doi: 10.1371/journal.pone.0186600 (PMC5648225; doi:10.1371/journal.pone.0186600)
Supplement: S1 Table — (DOCX) [file pone.0186600.s011.docx]

**S1 Table. Sedimentary sequence of the Boseong fossil site.**

| **Main Petrology** | | Epiclastic, pyroclastic, and intermediate to acidic volcanic rocks |
| --- | --- | --- |
| **Sedimentary sequence**  **(in descending order)** | | Docheonri Rhyolite |
|  |  | Obongsan Brecciated Tuff (has the widest distribution in the local area) |
|  |  | Mudeungsan Flow |
|  |  | Pilbong tuff (or Pilbong Rhyolite) |
|  |  | Seonso Formation (fossiliferous sedimentary layer) |
|  |  | Seonso Conglomerate (fossiliferous sedimentary layer) |
|  |  | Lapilli tuff |
| **Fossiliferous sedimentary layers – Seoso Formation and Seonso Conglomerate** | **Petrology** | Primarily clastic |
|  |  | Conglomerates, sandstones, mudstones, and alternating sandstones and mudstones |
|  | **Inferred paleoenvironment** | Alluvial environments such as alluvial fan, meandering river, and braided river based on upwards-fining units |
|  |  | Semiarid paleoclimate based on calcic and vertic paleosols formed by alternating dry and wet periods |
|  | **Factors involved in egg and bone fossil preservation** | Rapid burial likely due to river floodplains |
|  |  | Sheetflooding considered as the dominant process |
|  |  | Calcic palaeosols may have also aided fossil preservation |
