# Supplementary material for: Correlative microscopy of the constituents of a dinosaur rib fossil and hosting mudstone: Implications on diagenesis and fossil preservation
Source: PLoS One. 2017 Oct 19;12(10):e0186600. doi: 10.1371/journal.pone.0186600 (PMC5648225; doi:10.1371/journal.pone.0186600)
Supplement: S2 Table — (DOCX) [file pone.0186600.s012.docx]

**S2 Table. The interplanar spacings and chemistry of clay phases from FIB-milled samples.**

| **FIB Samples** | | **Interplanar Spacing** | | **Chemistry** | | **Interplanar Spacing** | **Chemistry** | | **Interplanar Spacing** | **Chemistry** | |
| --- | --- | --- | --- | --- | --- | --- | --- | --- | --- | --- | --- |
|  |  | **≈10 Å** | **≈20 Å** | **Elements** | **Scan Type** | **12~14 Å** | **Elements** | **Scan Type** | **14 ~15 Å** | **Elements** | **Scan Type** |
| FIB 1 | -1 (Boundary) | - | - | - | - | - | - | - | 4 | Si, Mg, Al, Fe, K | A(8), M(1) |
|  | -2 (Boundary) | 5 | - | Si, Al, K, Mg, Fe | A(2) | - | - | - | - | - | - |
|  | -3 (Boundary) | 2 | - | Si, Al, K, Mg, Fe | A(3), M(3) | - | - | - | - | - | - |
|  | -4 (Mudstone) | 10 | - | Si, Al, K, Mg, Fe | A(12), M(3) | - | - | - | - | - | - |
|  | -5 (Bone) | 3 | 2 | Si, Al, K, Mg, Fe | A(4), M(2) | - | - | - | - | - | - |
| FIB 2 | -1 (Boundary) | 2 | - | Si, Al, K, Mg, Fe | A(2) | - | - | - | - | - | - |
|  | -2 (Boundary) | 3 | - | Si, Al, K, Mg, Fe | A(3) | - | - | - | - | - | - |
|  | -3 (Boundary) | 2 | - | Si, Al, K, Mg, Fe | A(2) | - | - | - | - | - | - |
|  | -4 (Mudstone) | 10 | - | Si, Al, K, Mg, Fe | A(14) | - | - | - | 1 | Si, Mg, Al, Fe  Si, Al, Mg, Fe, K | A(3) |
|  | -5 (Bone) | 1 | - | Si, Al, K, Mg, Fe | M(2) | - | - | - | - | - | - |
| FIB 3 | -1 (Boundary) | - | - | - | - | - | - | - | - | Si, Al, Mg, Fe, K | M(1) |
|  | -2 (Boundary) | - | - | - | - | 5 | Mg, Si, Al, Fe  Si, Mg, Al, Fe, K | A(6) | 2 | Mg, Si, Al, Fe | A(1), M(1) |
|  | -3 (Boundary) | 7 | - | Si, Al, K, Mg, Fe | A(4), M(1) | - | - | - | - | Si, Al, Mg, Fe, K | A(2) |
|  | -4 (Mudstone) | 5 | - | Si, Al, K, Mg, Fe | A(4), M(1) | - | - | - | 4 | Si, Al, Mg, Fe, K | A(4) |
|  | -5 (Bone) | - | - | Si, Al, K, Mg, Fe | A(2), M(1) | - | - | - | - | - | - |
| FIB 4 | -1 (Cross) | 13 | - | Si, Al, K, Mg, Fe | A(9), M(1) | - | - | - | - | - | - |
|  | -2 (Plane) | 8 | - | Si, Al, K, Mg, Fe | A(8), M(1) | - | - | - | - | - | - |
| FIB 5 | -1 (Mudstone) | 8 | 2 | Si, Al, K, Mg, Fe | A(7) | - | - | - | 2 | Si, Mg, Al, Fe, K  Si, Al, Mg, Fe, K | A(2) |
|  | -2 (Boundary) | - | - | Si, Al, K, Mg, Fe | A(1), P(8) | - | - | - | - | Si, Al, Mg, Fe, K | P(2) |
|  | -3 (Bone) | 8 | 1 | - | - | - | - | - | - | - | - |
| FIB 6 | -1 (Boundary) | 9 | - | Si, Al, K, Mg, Fe | A(9) | - | - | - | - | - | - |
|  | -2 (Bone) | 12 | - | Si, Al, K, Mg, Fe | A(12) | - | - | - | - | - | - |
| FIB 8 | (Boundary) | 1 | - | Si, Al, K, Fe, Mg | M(1) | - | - | - | 1 | Si, Mg, Al, Fe, K | M(1) |
| TOTAL | | 109 | 5 | 122 | | 5 | 6 | | 14 | 26 | |
|  |  | 114 | |  |  |  |  |  |  |  |  |

EDS Scan Type: A = area, M = map, P = point.

FIB 7 was focused only on apatite arrangement analysis.
